# High brain network system segregation is differentially linked with cognitive performance across the life span

**DOI:** 10.1162/NETN.a.542

**Published:** 2026-04-22

**Authors:** Cameron N. Calder, Carl Helmick, Javeria Ali Hashmi

**Affiliations:** Department of Medical Neuroscience, Dalhousie University; Psychiatry Brain Imaging Laboratory, Dalhousie University; Department of Anesthesia, Pain Management, and Perioperative Medicine, Dalhousie University; Nova Scotia Health Authority

**Keywords:** Cognitive aging, Cognition, Resting-state fMRI, Functional connectivity, Functional brain networks, System segregation

## Abstract

Healthy aging is marked by changes in both cognitive performance and the organization of brain networks. Declines in cognition have been linked to reductions in system segregation (SS), as older adults typically exhibit less segregated functional networks than younger adults. While lower segregation has been associated with diminished cognitive abilities, it remains unclear how individual variability in SS contributes to cognitive outcomes across the lifespan. Here, we examine relationships between SS and three cognitive domains (semantic, executive, episodic memory) using resting-state fMRI data from 179 younger (18–29 years) and 117 older adults (60–89 years). SS was measured globally and for specific networks using Schaefer’s 7-network parcellation. Our findings confirmed a global age-related reduction in SS, particularly impacting the somatomotor, ventral attention, and frontoparietal networks. This reduction in global SS mediated negative effects of age group on semantic and executive performance. When examining younger and older groups separately, we found that higher SS was associated with better semantic performance in both groups, while observing a similar positive association with executive performance only in older adults, suggesting that executive function becomes increasingly dependent on preserved network architecture with age. Maintaining SS may therefore be critical for supporting healthy cognitive aging.

## Author Summary

As we age, functional brain networks become less segregated, and this change is linked to age-related cognitive decline. However, it remains unclear whether low segregation impacts cognition similarly across age groups. In this study, we used resting-state fMRI to assess system segregation and its relation to executive, semantic, and episodic performance in younger and older adults. Consistent with prior research, we observed system segregation was lower in older adults. Furthermore, system segregation was broadly linked to semantic performance, but was associated with executive ability specifically in older adults. Network-level analyses revealed more detail on how segregation changes with age and is differentially linked to cognition across age groups. These findings emphasize system segregation as a key metric for understanding, and potentially optimizing, cognitive aging.

## INTRODUCTION

Throughout their lifespan, healthy individuals undergo age-related changes in brain function and cognition (Raz & Rodrigue, 2006; Salthouse, 2010). As the global population ages, maintaining cognitive function and preventing age-related diseases are crucial for quality of life, and empowering older adults to remain active in society (United Nations, Department of Economic and Social Affairs, Population Division, 2019). Cognitive decline occurs with healthy aging even without degenerative pathology (Krivanek et al., 2021) and is typically associated with declines in episodic memory and executive functioning despite stability or improvement in semantic (crystallized) intelligence (Spreng & Turner, 2019; Tucker-Drob et al., 2019). These changes must be clearly mapped brain network topology to facilitate effective prevention and management strategies. Furthermore, understanding the functional changes that occur in the aging healthy brain may provide insight into what goes awry in age-related neurological diseases.

Higher-order cognitive processes are widely distributed across the brain, requiring complex cooperation between various areas (Mesulam, 1990; Seeley et al., 2009; Wig et al., 2011). Neuroimaging studies have shown that the brain consists of functionally significant and highly internally connected subnetworks, linked to each other relatively sparsely (Baum et al., 2017; Khan et al., 2022; Rubinov & Sporns, 2010; van den Heuvel & Sporns, 2013). One approach for understanding neurological correlates of cognition is to investigate the integrity and interactions of these functionally relevant networks by examining patterns of resting-state functional connectivity. Among network analysis techniques used to study cognitive aging (see Deery et al., 2023), system segregation (SS) has emerged as a particularly robust marker given its consistent declines with age (Chong et al., 2019; Deery et al., 2023) and sensitivity to age-related cognitive changes (Chan et al., 2014; Madden et al., 2020). SS is a measure of the balance in the connectivity within and between subnetworks (Chan et al., 2014). From a general theoretical perspective, SS tracks the balance between the capacity for specialized functioning that segregation allows and the opposing ability to integrate information across systems (Aristi et al., 2022; Bullmore & Sporns, 2012; Hashmi et al., 2017). This balance, previously linked with individual differences in higher-order cognitive functions (Cohen & D’Esposito, 2016; Mišić & Sporns, 2016), may become disrupted in aging, contributing to cognitive decline.

Current literature supports that SS decline with aging is detrimental to cognitive ability (Chan et al., 2014; Kong et al., 2020; Zhang & Diaz, 2023). However, several details concerning this association remain unclear. While studies in younger adults suggest that greater functional network integration (consistent with lower SS) can predict improved performance on certain cognitive tasks (Cohen & D’Esposito, 2016; Wang et al., 2021; Yue et al., 2017), the aging literature paints a different picture. Reduced SS has been broadly associated with age-related declines across multiple cognitive domains (Chan et al., 2014; Kong et al., 2020; Stumme et al., 2020; Zhang & Diaz, 2023). However, these associations are not always consistent. For example, while some studies report SS-related declines in executive function (Chong et al., 2019; Kong et al., 2020; Madden et al., 2020; Raykov et al., 2024), episodic memory (Chan et al., 2014; Kong et al., 2020; Pedersen et al., 2021), and verbal fluency (Zhang & Diaz, 2023), other studies find no effect of segregation on one or more of these domains (Chan et al., 2014; Chong et al., 2019; Kong et al., 2020).

Importantly, age-related decline in SS is not uniform across individuals (Chong et al., 2019; Pedersen et al., 2021). The trajectory of cognitive decline shows a similar variability, with some individuals maintaining cognition more than others (Salthouse, 2019; Spreng & Turner, 2019; Tucker-Drob et al., 2019). Thus, if declining SS produces effects on cognition that are distinct from those of stable individual differences in SS, we would expect a different pattern of associations between SS and cognition across age groups and a tighter coupling between in older adults. Disentangling these patterns by separately examining individual differences within each age group may help clarify the specific cognitive benefits of retaining SS in older individuals. To our knowledge, no studies have yet examined the relationship between SS and multiple domains of cognition while distinguishing younger and older adults. Understanding this variability may ultimately inform strategies to promote cognitive resilience by preserving SS in aging populations.

In the present study, we used a publicly available dataset (Spreng et al., 2022) containing two age groups: a younger adult group (18–34 years), with ages ranging around the developmental peak of SS (Chan et al., 2014; Gu et al., 2015; He et al., 2019), and an older adult group (60–89 years) with ages ranging past the point of accelerated SS decline (Pedersen et al., 2021). Using these two clearly defined age groups, we independently evaluated the effects of SS on cognition in younger and older adults. We hypothesized that SS would relate differently to cognitive performance in older compared with younger adults, with low SS having a stronger negative impact in older adults.

## METHODS

### Participants

Resting-state fMRI (rs-fMRI) and cognitive testing data were obtained from the publicly available database “Goal-Directed Cognition in Older and Younger Adults” provided by Spreng et al. (2022). The sample was well suited to our purposes, having been designed to provide a reliable dataset for examining the role of functional brain networks in cognitive aging (see Setton et al. [2022] for their preliminary analysis) and including two clearly defined age groups. Specifically, they collected a younger adult sample (18 to 34 years) and a distinct older adult sample (60 to 89 years), recruited from Ithaca, New York (Site 1, *n* = 235) and Toronto, Canada (Site 2, *n* = 61). Inclusion criteria for this study included being right-handed and having normal or corrected-to-normal vision and not being depressed or cognitively impaired, as assessed by appropriate clinical scales (Beck Depression Inventory [Beck et al., 1996], Geriatric Depression Scale [Yesavage et al., 1982], Mini-Mental State Examination [Folstein et al., 1975], NIH fluid cognition score [Gershon et al., 2013]). Furthermore, five participants from the open-access database were not included in this study due to errors during data extraction and preprocessing (sub-33, sub-35, sub-117, sub-284, sub-301). All participants in this dataset gave informed consent under the guidelines established by the Institutional Review Boards at Cornell University and York University (Spreng et al., 2022).

### Cognitive Performance

The majority of measures analyzed in the present study were taken from the NIH Toolbox of Cognition (Gershon et al., 2013), while auxiliary cognitive measures included Verbal Paired Associates from the Wechsler Memory Scale–Fourth Edition (Wechsler, 2009), the Associative Recall Paradigm (Brainerd et al., 2014), Shipley-2 Vocabulary (Shipley et al., 2009), and Trail Making Test B-A (Reitan, 1958). A trained psychometrist collected all in-lab behavioral data to ensure consistent measurement. Paper-and-pencil tests were digitized by two researchers to guarantee accuracy, while attention checks during online data collection ensured that inattentive participants (failure of four or more attention checks) were excluded. Participants who gave uniform responses on three or more different questionnaires were excluded entirely, as were those with more than 15% missing behavioral data (Spreng et al., 2022).

Composite indexes for episodic memory, semantic memory, and executive function were constructed with selected tests from the NIH Cognition toolbox and all auxiliary cognitive measures, using the average of Z-transformed scores on the tests that were selected to compose a given index (Spreng et al., 2022). The episodic index included the NIH Cognition Picture Sequence memory task scored using item response theory (IRT), and unadjusted total scores (items remembered) on the NIH Cognition Rey Auditory Verbal Learning task (Slotkin et al., 2012), the Associative Recall test (Brainerd et al., 2014), and immediate recall, delayed recall, and delayed free recall scores on the Verbal Paired Associates task (Wechsler, 2009). The semantic index was composed of unadjusted score (total correct answers) on the Shipley Vocabulary task (Shipley et al., 2009), and IRT-scored NIH Cognition Picture Vocabulary and Oral Reading Recognition (Slotkin et al., 2012). The executive index included total unadjusted scores on the NIH Cognition Flanker Inhibitory Control and Attention task and the NIH Cognition Dimensional Change Card Sort task, both scored using reaction time and accuracy (Slotkin et al., 2012), and the NIH Cognition List Sort Working Memory, scored on accuracy alone, (Slotkin et al., 2012). Also included in the executive index was inverse score (inverted so that higher scores reflect better performance) on the Trail Making Task B-A (Reitan, 1958; Spreng et al., 2022). If some, but not all, of the test scores composing a composite metric were missing, the index was still computed using age-group means to fill in the missing test scores.

### Imaging Acquisition

Neuroimaging data were collected at two sites: using a 3 T Siemens TimTrio MRI scanner with a 32-channel head coil at the York University Neuroimaging Center in Toronto and a 3 T GE Discovery MR750 MRI scanner with a 32-channel head coil at the Cornell Magnetic Resonance Imaging Facility. At both sites, and for each participant, a T1 anatomical scan and two resting-state BOLD functional scans were collected. At both the Cornell Magnetic Resonance Imaging Facility and the York University Neuroimaging Center, all scans were conducted by a skilled MR technician following the same standardized protocol. This approach guaranteed uniform data collection practices across sites, which further included visual inspections for proper coverage, continuous quality monitoring, and verification of participant alertness (Spreng et al., 2022).

T1 anatomical scans on the Siemens scanner were obtained using a T1-weighted volumetric magnetization prepared rapid gradient echo sequence (TR = 1,900 ms; TE = 2.52 ms; 9° flip angle; 1-mm isotropic voxels, 192 slices, 4 min 26 s) with 2x acceleration and generalized auto-calibrating partially parallel acquisition (GRAPPA) with an iPAT acceleration factor of 2. Anatomical scans on the GE were acquired with a T1-weighted volumetric magnetization prepared rapid gradient echo sequence (TR = 2,530 ms; TE = 3.4 ms; 7° flip angle; 1 mm isotropic voxels, 176 slices, 5 min 25 s) with sensitivity encoded 2× acceleration.

Each of the two resting-state BOLD functional scans were 10 min and 6 s long The scanner bay was dimly lit. Participants were instructed to stay as still as possible during the scan with their eyes open while blinking and breathing normally. Rs-fMRI data were acquired with a multi-echo EPI sequence on GE (TR = 3,000 ms; TE1 = 13.7 ms, TE2 = 30 ms, TE3 = 47 ms; 83° flip angle; matrix size = 72 × 72; FOV = 210 mm; 46 axial slices; 3 mm isotropic voxels; 204 volumes, 2.5× acceleration with sensitivity encoding) and Siemens (TR = 3,000 ms; TE1 = 14 ms, TE2 = 29.96 ms, TE3 = 45.92 ms; 83° flip angle; matrix size = 64 × 64; FOV = 216 mm; 43 axial slices; 3.4 × 3.4 × 3 mm voxels; 200 volumes, 3× acceleration and GRAPPA encoding) scanners. Participant 149 had 206 volumes collected instead of 204, which was accounted for in preprocessing. The original rs-fMRI dataset was published for open access on Open Neuro (Spreng et al., 2021).

### Quality Assurance

Several quality assurance checks were conducted prior to data release. Unsuccessful co-registration, framewise displacement (FD), temporal signal-to-noise ratio (TSNR), and BOLD dimensionality were quantified for each participant’s scan, and their data were excluded if these metrics fell outside of acceptable ranges (FD > 0.50 mm coupled with denoised DVARS > 138; TSNR < 50; < 10 BOLD components retained after denoising). Of note was that BOLD dimensionality, the number of BOLD components identified in an independent components analysis of the fMRI time series, was significantly greater in younger participants than in the older group. The published dataset used in this paper was without significant neuroanatomical abnormalities, and all participant surfaces were found to have a Euler number of 2 (indicating no holes or defects). Further details on quality assurance procedures can be found in Spreng et al. (2022).

### Preprocessing

Preprocessing of anatomical and functional MRI data was conducted using fMRIPrep 22.0.1 (Esteban et al., 2019), which utilizes Nipype 1.8.4 (Gorgolewski et al., 2011). In brief, preprocessing of T1-weighted anatomical data included correction for field inhomogeneity bias, skull stripping, segmentation of brain tissues, and spatial normalization to the Montreal Neurological Institute (MNI) 152 standard template. For each of the two resting-state functional runs, a BOLD reference volume was created, followed by head motion estimation, spatiotemporal filtering (6 mm FWHM, 0.08–128 Hz), correction for field inhomogeneity-induced distortions, slice-timing correction, standardization, and detrending. The BOLD reference was then co-registered to the high-resolution T1-weighted anatomical reference using boundary-based registration. Confound time series were derived based on head motion metrics (e.g., FD, DVARS) and global signals (GSs) from cerebrospinal fluid, white matter, and whole-brain GS. Preprocessed BOLD time series were resampled to the standard MNI152 template space (152NLin2009cAsym, https://nist.mni.mcgill.ca/icbm-152-nonlinear-atlases-2009/). Time series were extracted using the Schaefer et al. (2018) parcellation (200 ROI, 7-network) from TRs 5 to 200 (to account for non-steady-state outliers and discrepancies in Participant 149’s runtime) and concatenated across the two resting-state runs. To account for between-subject differences in head motion, we quantified fMRI movement as the percentage of flagged volumes (standardized DVARS > 1.5 or FD > 0.5 mm; Power et al., 2012). This percentage was computed for each scan and averaged across both scans for use as a between-subject motion covariate.

### System Segregation Analysis

Whole-brain binarized SS was assessed using a graph theory approach. A 200 × 200 adjacency matrix was constructed for each participant by computing zero-lag Pearson’s linear correlations across the 392 volume BOLD time series. Negative values and diagonal entries were removed from the correlation matrices, consistent with previous best practices in the application of graph theory to BOLD data (Rubinov & Sporns, 2010), and to simplify the interpretation of our results in relation to standard functional network definitions (Schaefer et al., 2018). Each matrix was thresholded and converted into a binarized adjacency matrix to isolate only the significant functional connections and minimize the impact of physiological or experimental noise (van Wijk et al., 2010). Thresholding was performed using relative correlation thresholds, ranging from 0.05 to 0.50 in increments of 0.05, such that at a threshold of 0.05, only the highest 5% of Pearson correlation coefficients (*r* values) form an edge of the binarized adjacency matrix, while at the 0.50 threshold, the strongest 50% of *r* values would be included as a surviving edge (Aristi et al., 2022; Bassett et al., 2008). Given the lack of consensus in choosing an optimal threshold, this range of thresholds was used to test for consistency of the results at different network costs. While more inclusive thresholds allow for spurious connections, leading to noisier data, more exclusive thresholds can remove weak but functionally significant connections, leading to a less sensitive characterization of the system’s interactions (van den Heuvel et al., 2017; Zalesky et al., 2016). To account for spurious correlations given the range of thresholds tested, we set a minimum of significance at three thresholds for results to be considered significant (Pak & Hashmi, 2023). We defined functional networks using the 7-network parcellation by Schaefer et al. (2018). Binarized SS was defined as the difference between within-network and between-network connectivity, proportional to within-network connectivity. SS was calculated by the following formula:

$$\text{System}\;\text{Segregation}=\frac{B_{w}-B_{b}}{B_{w}}$$

where *B*_*w*_ is the proportion of within-network edges that survived thresholding, and *B*_*b*_ is the proportion of between-network edges that survived thresholding. The code for calculating system segregation from a connectivity matrix was sourced from the study of Chan et al. (2014). Since previous work suggests cognition is more sensitive to subnetwork segregation (Chong et al., 2019), we used the method described above to calculate segregation scores for each network (visual, somatomotor, dorsal attention, ventral attention, limbic, frontoparietal, and default mode) in the Schaefer 7-network parcellation (Schaefer et al., 2018). In these subnetwork segregation measures, possible within-network edges were defined as all ROI pairs within the given network, while possible between-network edges were defined as all ROI pairs consisting of one ROI in the given network and one ROI in any other network. By contrast, in our global SS measure, possible within-network edges included all ROI pairs with the same network membership, whereas possible between-network edges included all ROI pairs with different network memberships.

## RESULTS

### Demographics

Our final sample included 179 healthy younger adults (mean age = 22.6 years, *SD* = 3.3, range = 18–34) and 117 healthy older adults (mean age = 68.7 years, *SD* = 6.5, range = 60–89) recruited from Ithaca, New York (Site 1, *n* = 235) and Toronto, Canada (Site 2, *n* = 61). Comparisons revealed several significant demographic differences between our age groups, including site, years of education, as well as racial and ethnic background. Specifically, our older adult group was more predominantly non-Hispanic White and included a higher proportion of participants from Toronto than our younger group. No significant differences were observed between age groups in sex distribution (Table 1).

**Table 1.** Demographic information from the open-access dataset: “Goal-Directed Cognition in Younger and Older Adults”

	Younger Adults	Older Adults	Test Statistic	Sig. (*p*)
Sex (% female)	57.9	54.7	*χ*^2^ = 0.23	.630
Site (% Toronto)	15.1	29.1	*χ*^2^ = 8.45	.004
Race				
White (%)	56.4	91.5	*χ*^2^ = 27.61	.001
Asian (%)	17.9	1.7	*χ*^2^ = 21.79	.001
Black (%)	8.4	2.6	*χ*^2^ = 5.43	.020
Other (%)	3.9	2.6	*χ*^2^ = 0.77	.401
Not provided (%)	14.5	1.7	—	—
Ethnicity				
Hispanic (%)	9.5	1.7	*χ*^2^ = 8.27	.004
Not provided (%)	19	9.4	—	—
Education (years)			*U* = 5476	<.001
Range	12–24	12–24		
*M* (*SD*)	15.21 (1.92)*	17.26 (2.91)*		
fMRI movement (%)			*U* = 8963	.036
Range	0.98–49.75	0.98–60.50		
*M* (*SD)*	7.96 (8.56)*	11.63 (12.85)*		

*Note*. Chi-square tests were used to assess age-group differences in categorical variables, and Mann–Whitney *U* tests were used for years of education to account for unequal variance between groups. * Levene’s test indicated homogeneity of variance was violated for years of education (*F*(1, 294) = 29.77, *p* < 0.001) and fMRI movement (*F*(1, 294) = 14.93, *p* < 0.001) between age groups.

Only sex and within-group age were included as covariates in the following primary analyses. These were essential to control for, given the evidence of continuous age-related changes in SS and cognition, as well as prior research demonstrating sex differences in both functional connectivity and cognitive performance (Miller & Halpern, 2014; Zhang et al., 2018). Differences in racial and ethnic composition were not corrected, given the small number of non-White participants in the older cohort; this approach is consistent with prior studies of system segregation in aging, though it remains a potential limitation.

Site effects were addressed in a separate sensitivity analysis, given uncertainty about how site correction may impact the analysis. Specifically, it was unclear whether site differences simply reflect natural population variance, especially considering the measures taken to minimize data collection differences between sites (Spreng et al., 2022). However, fMRI movement was substantially higher in Site 2, meaning that site and motion were highly collinear. This further supported analyzing the sites separately, particularly when correcting for motion (Van Dijk et al., 2012).

### Differences in System Segregation Between Age Groups

**Figure 1.**
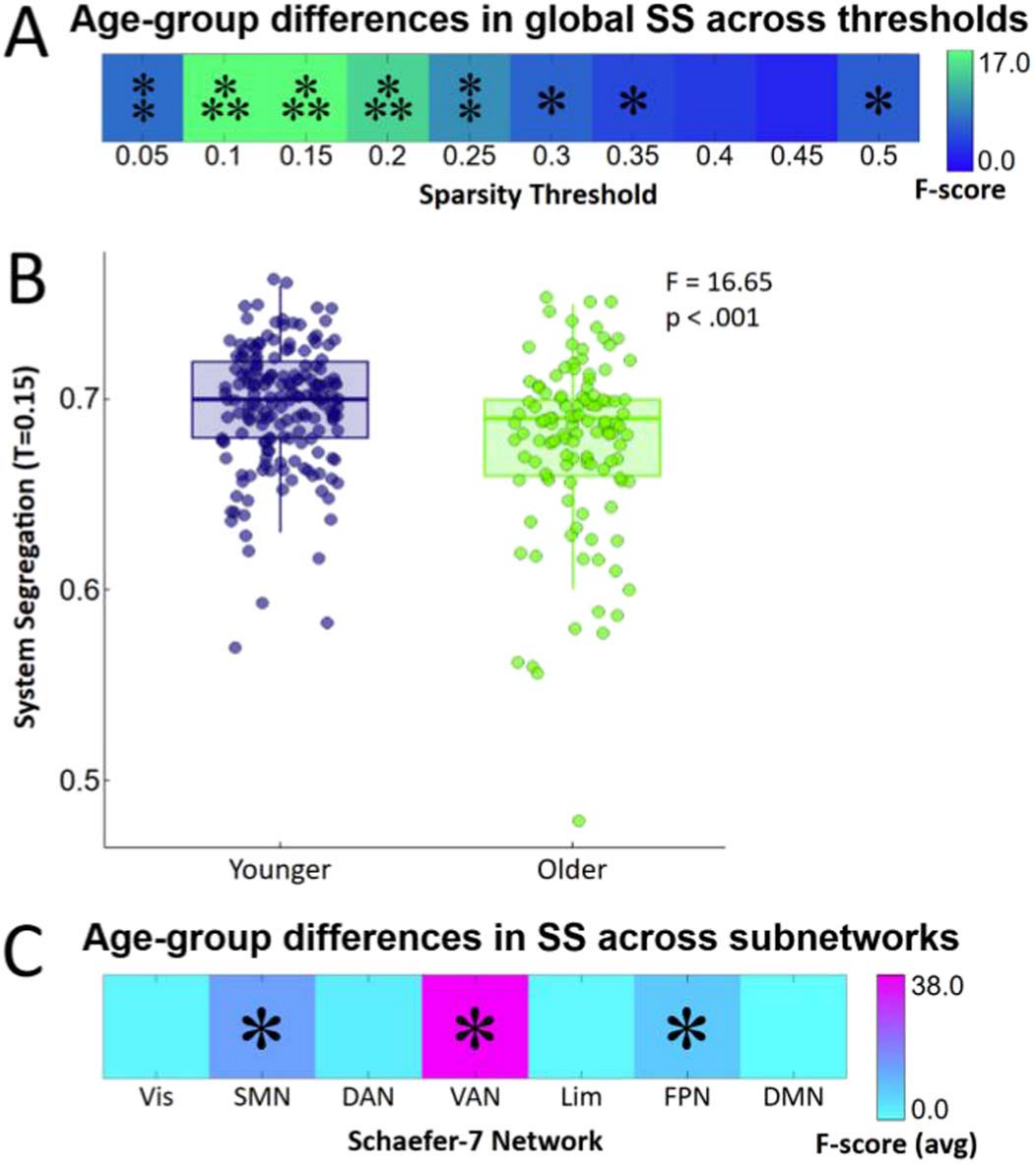
Differences in global and subnetwork SS in older and younger adults. (A) Global SS calculated across a range of sparsity thresholds shows significant differences between younger (18–29 years; *n* = 179) and older (60–89 years; *n* = 117) adults as revealed from a two-way ANOVA with sex included as a covariate. (B) Univariate scatter plot displaying group differences in global SS between younger and older adults with sparsity threshold (T) set at 0.15. (C) Aging-related reduction in SS is specific to the somatomotor (across all thresholds except for T = 0.45), ventral attention (across all thresholds) and FPNs (across all thresholds except for T = 0.05). The heatmap displays F-scores averaged across sparsity thresholds for each subnetwork as revealed from a two-way ANOVA with sex included as a covariate. Asymptotically approximated *p*-scores were calculated for each threshold with network abbreviations are as follows, Vis: Visual network, SMN: Somatomotor network, DAN: Dorsal attention network, VAN: Ventral attention network, Lim: Limbic network, FPN: Frontoparietal (control) network, DMN: Default mode network. ***, **, and * indicate significance at the 0.001, 0.01, and 0.05 levels, respectively, in Figure 1A. † indicating significance at our predefined minimum of three sparsity thresholds in Figure 1C.

We observed that global SS showed greater variance and lower values in older adults compared with younger adults. Levene’s test of homogeneity of variance failed across 9/10 sparsity thresholds, with the older age group showing greater variance in global SS than the younger group (Table S1 and Figure S1A in Supporting Information). Given the significance of Levene’s test, the rank-ordered two-way analysis of variance (ANOVA), with sex corrected as a covariate, was used to detect group differences in SS between younger and older adults with *α* = 0.05 and effects only considered significant if observed over three or more sparsity thresholds. Our analysis revealed a significant reduction in global SS in the older age group across a range of 8/10 sparsity thresholds (Table 2; Figure 1A).

**Table 2.** Significant age-group differences in global and subnetwork SS

Network	Sig. Sparsity Thresholds	*F*(1, 294)	Sig. (*p*)	Higher Segregation
Global	.05–.35, .50	4.67–16.76	< .001–.031	Younger
SMN	.05–.40, .50	4.99–29.81	< .001–.026	Younger
VAN	.05–.50	23.04–53.42	< .001 for all	Younger
FPN	.10–.50	4.53–12.93	< .001–.034	Younger

*Note*. The statistics reported here were calculated using rank-ordered two-way ANOVAs, with age group and sex included as independent variables. Ranges of F-scores and *p* values only include those sparsity thresholds which were significantly different between younger and older adults. See Table S3 in Supporting Information for individually reported significant thresholds, and Figure 1 for a visualization of these results. Network abbreviations are as follows, Vis: Visual network, SMN: Somatomotor network, DAN: Dorsal attention network, VAN: Ventral attention network, Lim: Limbic network, FPN: Frontoparietal (control) network, DMN: Default mode network.

When examining SS on a subnetwork level, we first observed that Levene’s test was significant across 31/70 subnetwork specific segregation measures, with the older age group showing greater variance than the younger group (Supporting Information Table S1 and Figure S2). Thus, the rank-ordered two-way ANOVA, with sex corrected as a covariate, was used to detect age-group differences in subnetwork segregation. As summarized in Table 2, significant group differences were found across 9/10 thresholds in the somatomotor network (SMN), all thresholds in the ventral attention network (VAN), and 9/10 thresholds in the frontoparietal network (FPN). In summary, as expected from previous literature, SS was significantly lower in our older adult group than our younger adult group. Furthermore, we demonstrate that these changes are related to lower segregation primarily in higher-order/association networks (VAN, FPN) but also in the SMN (Figure 1C).

To verify the robustness of these findings, we performed robust regression with age group as a binary predictor. Results were consistent with the rank-ordered tests, showing a significant negative association between age group and global SS across 7/10 thresholds. At the subnetwork level as well, robust regression confirmed that decreases in SS with age were most pronounced in the VAN (all thresholds), FPN (9/10 thresholds), and SMN (6/10 thresholds; Supporting Information Table S6).

We also examined the effect of age on SS as a continuous variable within each age group. Shapiro–Wilk tests indicated normality was violated for global SS at 9/10 thresholds in younger adults and at all thresholds in older adults (Supporting Information Figure S1A and Table S2). Furthermore, across 7 subnetworks with 10 thresholds each, SS violated normality in 54/70 measures in the younger group, and 63/70 SS measures in the older group (Supporting Information Figure S2 and Table S2). Thus, we used Spearman’s partial correlations, controlling for sex, to test associations between age and SS within groups.

Global SS was not significantly correlated with age in either group across our cutoff of three or more sparsity thresholds. However, at the subnetwork level, segregation of the limbic network (4/10 thresholds) and the default mode network (DMN; 4/10) increased with age in younger adults (Supporting Information Figure S3B–C), whereas segregation of the SMN (4/10), VAN (5/10), and limbic network (6/10) decreased with age in older adults (Supporting Information Figure S3D–F). We also tested associations between within-group age and cognition. No significant effects were observed in younger adults (executive: *r*_*s*_ = −0.074, *p* = 0.353; semantic: *r*_*s*_ = −0.033, *p* = 0.678; episodic: *r*_*s*_ = −0.145, *p* = 0.068) while in older adults, age correlated negatively with executive (*r*_*s*_ = −0.247, *p* = 0.007) and episodic performance (*r*_*s*_ = −0.346, *p* < 0.001) and positively with semantic performance (*r*_*s*_ = 0.239, *p* = 0.010). These findings justify the inclusion of within-group age as a covariate in later analyses.

To test whether SS adds predictive value beyond cognition in distinguishing younger from older adults, we trained two logistic regression models: one with cognitive features alone (cognition-only) and another with both cognitive features and global SS across sparsity thresholds (cognition+SS). Only participants with complete cognitive data were included (*n* = 278). Both models were split identically into a training set and a held-out test set (70/30). Logistic regression models were trained using nested five-fold cross-validation with regularization strength tuned in the inner loop (L2 penalty). To statistically compare models, performance metrics were computed and tested with paired *t* tests across identical outer folds.

Classification performance was nearly identical between cognition-only and cognition+SS (Supporting Information Table S7), with no significant differences in AUC (*p* = 0.51), accuracy (ACC) (*p* = 0.62), or balanced accuracy (*p* = 0.79). Consistent with this pattern, a random forest classifier trained on the same data showed that adding SS to cognition produced slightly weaker, though statistically comparable, performance (Supporting Information Table S7). On the independent test set, cognition-only achieved near-perfect classification (Supporting Information Table S8; *AUC* = 1.000, *ACC* = 0.982), indicating no overfitting. Adding SS did not improve generalization (*AUC* = 0.999, *ACC* = 0.982) and including sex alongside SS modestly reduced accuracy (*AUC* = 0.987, *ACC* = 0.929). Together, these results indicate that cognition alone nearly saturates classification performance, and global SS does not provide incremental predictive value for distinguishing younger from older adults.

### Relationship Between System Segregation and Cognition

Having established differences in SS and cognition between younger and older adults, we next examined the relationships between these variables within each age group. As in our within-group age analysis, deviations from normality in both global and subnetwork segregation measures (Supporting Information Table S2 and Figure S1A) indicated the use of nonparametric Spearman’s rank partial correlations. Sex and within-group age were included as covariates, and analyses were conducted separately within each age group.

**Figure 2.**
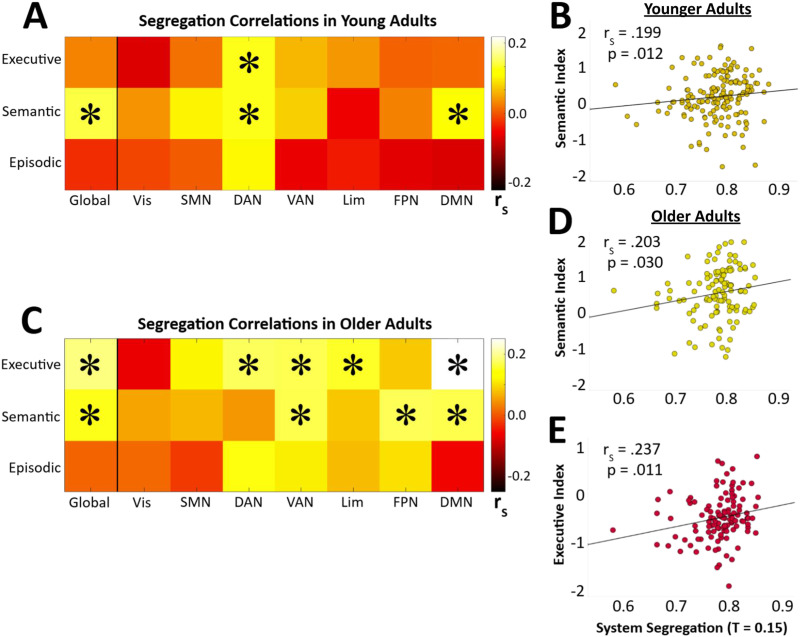
Higher SS is related to higher cognitive scores on a global and subnetwork specific level. (A) In younger adults (18–29 years; *n* = 161), individual differences in global SS correlated with semantic performance, and both semantic and executive performance were linked to SS on a subnetwork level. (B) Scatterplot displaying the relationship between global SS and semantic performance in younger adults. (C) Individual differences in system segregation correlated with executive and semantic performance both on global and subnetwork levels in older adults (60–89 years; *n* = 117). Heat maps show *r*_s_ values from Spearman’s partial correlations, corrected for age and sex, and averaged across all sparsity thresholds (including nonsignificant ones). (D) and (E) Scatterplots displaying the correlation between SS (sparsity threshold set at 0.15) in older adults with the executive index and semantic index, respectively. For all scatterplots, Spearman’s partial correlation coefficients (*r*_s_) and corresponding *p* values are reported, while the data points shown represent the raw, uncorrected values. * indicates group differences were significant at the 0.05 level for our predefined minimum of three sparsity thresholds.

In younger adults, global SS was positively correlated with semantic memory performance (6/10 thresholds), but not with other cognitive indices (Figure 2B). At the subnetwork level, we found positive correlations between segregation of the dorsal attention network (DAN) and both executive (5/10 thresholds) and semantic performance (5/10 thresholds), as well as between DMN segregation and semantic performance (3/10 thresholds; Table 3; Figure 2A). While some correlations, such as between episodic cognition and DMN segregation, trended negatively, these effects were not significant.

**Table 3.** Significant Spearman correlations (*r*_s_) between global and subnetwork SS and cognition in younger and older adults

Age group	Cognitive Domain	Network	Sig. Sparsity Thresholds	Correlation (*r*_*s*_)	Sig. (*p*)
Younger	Executive	DAN	0.20, 0.25, 0.35–0.45	0.159–0.165	0.038 to 0.046
	Semantic	Global	0.05–0.25, 0.35	0.159–0.199	0.012 to 0.045
		DAN	0.20–0.40	0.161–0.177	0.025 to 0.043
		DMN	0.05, 0.40, 0.45	0.157–0.173	0.029 to 0.048
Older	Executive	Global	0.05–0.30	0.199–0.237	0.011 to 0.033
		DAN	0.05–0.20, 0.30	0.196–0.241	0.009 to 0.036
		VAN	0.05–0.20	0.206–0.253	0.006 to 0.027
		Limbic	0.05–0.15	0.244–0.285	0.002 to 0.009
		DMN	0.05–0.50	0.201–0.319	< 0.001 to 0.032
	Semantic	Global	0.05–0.15	0.201–0.228	0.014 to 0.031
		VAN	0.05, 0.10, 0.20	0.185–0.209	0.025 to 0.048
		FPN	0.05–0.10, 0.20–0.25	0.206–0.241	0.009 to 0.027
		DMN	0.05–0.15	0.185–0.213	0.022 to 0.048

*Note*. Analyses were corrected for sex and within-group age using partial correlations. Reported ranges of *r*_s_ values and *p* values reflect only those sparsity thresholds showing significant (*α* = .05) correlations with the indicated cognitive index. See Figure 2 for a visualization of these results. Threshold-specific results are provided in Supporting Information Tables S4 and S5.

In older adults, global SS was positively correlated with both executive cognition (6/10 thresholds; Figure 2E) and semantic performance (3/10 thresholds; Figure 2D). These associations were reflected at the subnetwork level, where executive cognition was linked to segregation of the DAN (5/10 thresholds), VAN (4/10), limbic (3/10), and DMN (all thresholds). Semantic performance was again related to DMN segregation (3/10 thresholds), but also to the VAN (3/10) and FPN (4/10), rather than to the DAN. Episodic memory performance showed no relationship to global SS or subnetwork in either group.

Overall, these results indicate age-group differences in the association between SS and cognition, with semantic performance linked to SS in both groups, but executive performance associated with SS primarily in older adults. In addition, we note that on a subnetwork level, these correlations were exclusive to higher-order/association networks such as the DAN, VAN, limbic network, FPN, and DMN.

Weaker correlations between SS and executive cognition observed in younger adults may be attributable to the lower variance in SS previously reported in this group, which would make statistical associations harder to detect (Supporting Information Table S1). To further investigate this possibility, we first examined whether variance in cognitive performance was also restricted in the younger group. For executive and semantic performance, variance was slightly lower in the older group (executive: *σ* = 0.474; semantic: *σ* = 0.708) relative to the younger group (executive: *σ* = 0.543; semantic: *σ* = 0.770), although these differences were not statistically significant (executive: *F*(1, 275) = 1.405, *p* = 0.237; semantic: *F*(1, 275) = 0.321, *p* = 0.572). In contrast, episodic performance showed significantly lower variance in the younger group (*σ* = 0.533) compared with the older group (*σ* = 0.663; *F*(1, 275) = 12.785, *p* < 0.001; Supporting Information Figure S1B–D).

To account for potential false negatives arising from restricted range in SS among younger adults, we applied Thorndike’s Case II range-restriction correction across both samples (Sackett & Yang, 2000; Thorndike, 1947). Pooled variances were used to estimate the population variance, and a modified version of Thorndike’s correction was employed for correlations involving the episodic index to account for bivariate restriction (Alexander et al., 1985). Following correction, correlations between semantic performance and VAN SS in the younger group became statistically significant across multiple thresholds (T^0.05^: *r*_*adj*_ = 0.180, *p*_*adj*_ = 0.002; T^0.10^: *r*_*adj*_ = 0.150, *p*_*adj*_ = 0.010; T^0.20^: *r*_*adj*_ = 0.124, *p*_*adj*_ = 0.033). A significant relationship was also found between segregation of the SMN and semantic performance in the younger group (T^0.10–0.45^: *r*_*adj*_ = 0.124, *p*_*adj*_ = 0.033; *r*_*adj*_ = 0.194, *p*_*adj*_ = 0.001; *r*_*adj*_ = 0.216, *p*_*adj*_ < 0.001; *r*_*adj*_ = 0.205, *p*_*adj*_ < 0.001; *r*_*adj*_ = 0.181, *p*_*adj*_ = 0.002; *r*_*adj*_ = 0.173, *p*_*adj*_ = 0.003; *r*_*adj*_ = 0.147, *p*_*adj*_ = 0.011; *r*_*adj*_ = 0.142, *p*_*adj*_ = 0.014). No other significant changes in correlations between SS and executive or episodic performance in the younger group, or in any of the associations within the older group. Thus, restricted variance may contribute to the fewer observed associations between semantic cognition and SS in the younger group. However, reduced variance in SS does not appear sufficient to account for the absence of executive associations in younger adults.

To further investigate the relationship between age, SS, and cognition, we tested whether global SS mediates cognitive differences between younger and older adults. The mediation effect of SS on the executive, semantic and episodic indices relation to age group was tested using the PROCESS software package (*n* = 278) with 5,000 bootstrap samples (Hayes, 2018). Mediation analyses were run for each sparsity threshold. To account for within-group age differences, group-relative age was calculated for each participant as [(age − agegroupmin)/agegrouprange], using the participants respective age group as the reference for agegroupmin and agegrouprange, and set as a covariate alongside sex.

**Figure 3.**
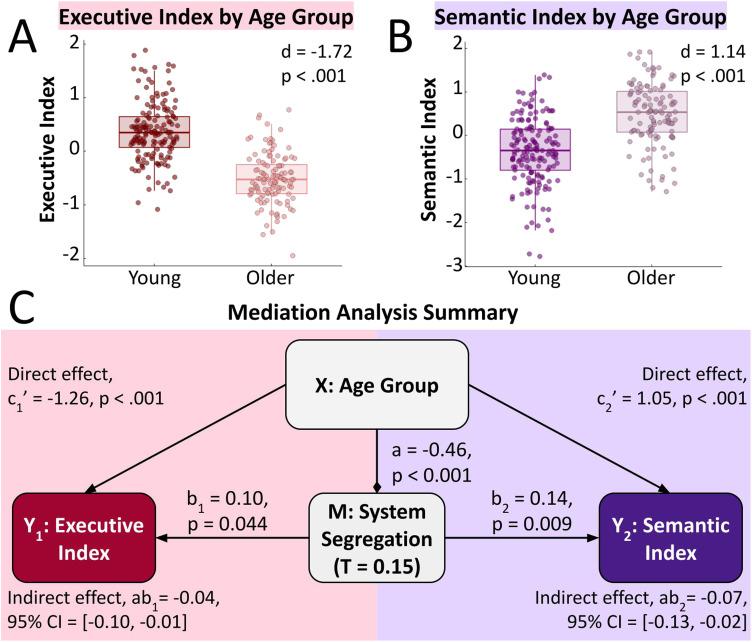
SS mediates small negative effects of age group on the semantic and executive indexes across all participants. For the semantic index, global SS acts as an inconsistent mediator (*n* = 278), with SS attenuating age-related improvements in semantic performance. In contrast, age-related decline in executive function was classically mediated by SS, exacerbating the decline of the direct effect of age. (A) Univariate scatter plot displaying group differences in the executive index between younger (18–29 years) and older (60–89 years) adults. (B) Univariate scatter plot displaying group differences in the semantic index between younger and older adults. (C) Diagram showing the full PROCESS mediation analysis on the effect of age group on semantic and executive indices with SS (T set at 0.15) as the mediator and group-relative age and sex as covariates. The arrows show predictive strength of X: age group and M: SS in regression models, with ♦ indicating a negative effect and ▸ indicating a positive effect. Age group was a binary predictor set as either younger or older group. Confidence intervals for overall effects were derived at a confidence level of 95% using 5,000 bootstrap samples. See Figure S4 in the Supporting Information for the null result of SS as a mediator of the episodic index.

The relationship between age group and SS (*a* in Figure 3C) is shared by the executive and semantic mediation models and was established to some extent in the previous section (Figure 1A). In the PROCESS model, age group as a predictor was tested using a simple linear regression with group-relative age and sex included as covariates. In this model, age group significantly negatively predicts SS at 7/10 sparsity thresholds (Table 4).

**Table 4.** Significant mediation pathways from a PROCESS mediation model evaluating global SS as a mediator of the effect of age group on cognition

Mediation step	Sig. Sparsity Thresholds	Standardized effect (*B*)	Sig.
Age group → SS (*a*)	0.05–0.40, 0.50	−0.268 to −0.465	*p* < 0.001 to *p* = 0.026
SS → executive performance (*b*_*1*_)	0.05–0.15	0.095 to 0.107	*p* = 0.022 to 0.044
Age group → SS → executive performance (*ab*_*1*_)	0.05–0.20	−0.038 to −0.050	95% CI = [−0.086, −0.001] to [−0.102, −0.008]
SS → semantic performance (*b*_*2*_)	0.05–0.25	0.108 to 0.154	*p* = .005 to .045
Age group → SS → semantic performance (*ab*_*2*_)	0.05–0.30	−0.032 to −0.072	95% CI = [−0.081, −0.001] to [−0.137, −0.023]

*Note*. Sex and group-relative age were included as covariates. Results are reported as ranges across all significant sparsity thresholds (*α* = .05) or as 95% bootstrap confidence intervals (5,000 samples), which did not include zero.

Finding the independent predictive power of age group (*c*_*1*_ in Figure 3C) and SS (*b*_*1*_ in Figure 3C) on the executive index was needed to complete the mediation model. Both pathways were tested in the PROCESS model with a multiple linear regression including SS and age group, and group-relative age and sex as covariates. We found that the effect of age group on the executive index was significant regardless of the sparsity threshold included (Figure 3A; *c*_*1*_ = −1.262, *p* < 0.001 in a model with T = 0.15). Additionally, SS positively predicted the executive index at 3/10 sparsity thresholds (Table 4). Given that paths *a, b*_*1*_, and *c*_*1*_ were all significant across our minimum number of thresholds, we can conclude that SS does act as a mediator between age group and the executive index. For these thresholds, estimated mediation effects (*ab*_*1*_ in Figure 3C) were weakly but significantly negative, consistent with a partial mediation effect (Table 4).

Using the aforementioned methodology in evaluating mediation for the semantic index, we found that the effect of age group on the semantic index was significant regardless of the sparsity threshold included (Figure 3B; *c*_*2*_ = 1.052, *p* < 0.001 in a model with T^0.15^). Additionally, SS positively predicts semantic index at 5/10 sparsity thresholds (Table 4). Paths *a, b*_*2*_, and *c*_*2*_ (Figure 3C) were all significant across our minimum number of thresholds, and the total estimated mediation effect (*ab*_*2*_) had a 95% confidence interval entirely below 0 at 6/10 thresholds, meaning that SS shows a significant partial inconsistent mediation effect on semantic performance (Table 4; Figure 3). This reflects inconsistent mediation given that effect of SS acts in opposition to the direct effect (MacKinnon et al., 2000), as opposed to a classical mediator that explains or enhances the total effect.

A mediation analysis using the method described above did find direct negative effects of age group on the episodic index (*B* = −1.223, *p* < .001 in a model with T^0.15^) but mediation through SS was not significant (Supporting Information Figure S4).

### Relationship of Sex With SS and Cognition

As a secondary analysis, we tested whether sex differences impacted SS and cognition. In the whole sample, we ran a two-way ANOVA with sex as the main factor, controlling for age group and including group-relative age as a covariate. Younger and older adults were also tested independently using one-way ANOVAs with group-relative age as a covariate. To account for the nonparametricity of SS, SS analyses were performed on rank-ordered data.

Sex had a moderate impact on both global and subnetwork SS, with females consistently showing higher SS. In the whole sample, females showed greater global SS (9/10 thresholds) and higher subnetwork segregation of the DAN (7/10 thresholds), VAN (5/10 thresholds), and DMN (all thresholds). When analyzing age groups separately, sex differences were observed in global SS (4/10 thresholds) and VAN segregation (3/10 thresholds) only in older adults, while both younger (all thresholds) and older females (8/10 thresholds) showed greater DMN segregation than their male counterparts. No sex differences were found in DAN segregation within age groups, likely reflecting limited statistical power in the smaller subsamples (Supporting Information Table S9). Sex also influenced cognition, particularly episodic and semantic performance. In the younger group, males performed better on semantic tasks (*F*(1, 275) = 5.01, *p* = 0.027), while females performed better on episodic tasks in both younger (*F*(1, 275) = 26.12, *p* < 0.001) and older adults (*F*(1, 275) = 9.61, *p* = 0.002), as well as in the whole sample (*F*(1, 275) = 26.12, *p* < 0.001; Supporting Information Table S10).

Finally, sex correction influenced the observed associations between SS and cognition. In sex-uncorrected models, DAN segregation correlated with episodic performance, suggesting that this relationship may be driven by sex differences. Conversely, the positive association between global SS and semantic performance in younger adults was no longer significant without sex correction, indicating potential confounding by sex (Supporting Information Tables S11–13).

### Sensitivity Analyses for Site and Motion Confounds

We noted slight differences in demographics, subnetwork SS, and cognition between the two recruitment sites in the “Goal-Directed Cognition in Younger and Older Adults” dataset. We previously noted that Site 2 (Toronto) included more older adults than Site 1 (Ithaca). Further analyses revealed that participants from Site 2 were significantly older within both younger (*t* = −2.91, *p* = 0.004) and older adults (Mann–Whitney *U* = 1002, *p* = 0.014; Supporting Information Table S14). They also showed significantly higher visual network SS (*U* = 5188, *p* < 0.001 at T^0.15^) and lower DMN SS (*t* = −3.75, *p* < 0.001 at T^0.15^) compared with Site 1 (Supporting Information Table S15). In terms of cognition, older adults from Site 2 demonstrated lower episodic (*t* = 2.55, *p* = 0.012) but higher executive performance (*t* = −3.86, *p* < 0.001), while younger adults from Site 2 showed reduced semantic performance (*t* = 2.98, *p* = 0.003; Supporting Information Table S16). Finally, we observed substantially higher fMRI movement in Site 2 than in Site 1 for both younger (Site 1: *M* = 6.01, *SD* = 5.97; Site 2: *M* = 18.94, *SD* = 12.13; *U* = 575, *p* < .001) and older adults (Site 1: *M* = 6.90, *SD* = 8.08; Site 2: *M* = 23.19, *SD* = 14.98; *U* = 363, *p* < .001; Supporting Information Table S14). This indicated noisier data from Site 2 and complicated the inclusion of fMRI movement as a covariate in the site-pooled analysis. To ensure our findings were robust to both site differences and head motion, we conducted sensitivity analyses excluding Site 2.

Following the removal of Site 2 (*n* = 62) and repeating the analysis without motion correction to match the primary model, results were largely unchanged. We observed the same pattern of reductions in global, SMN, VAN, and FPN segregation in older compared with younger adults. Correlations between SS and cognition were also consistent, with differences emerging only at the subnetwork level: DAN segregation was no longer correlated with executive performance in either group, while in older adults it became significantly correlated with episodic performance. Mediation effects also shifted slightly: after excluding Site 2, the pathway linking SS to executive cognition was no longer significant at three or more thresholds, due to path *b*_*1*_ at sparsity 0.15 falling just above significance. However, the total indirect effect still had a 95% confidence interval entirely below zero. This suggests that the loss of significance likely reflects reduced power to detect an already weak effect rather than site-related confounds. Overall, the sensitivity analysis did not contradict the primary results, with the main difference being the altered relationship between DAN segregation and cognition (Supporting Information Tables S17–19).

Adding fMRI movement as a covariate in the sensitivity analysis yielded results that closely matched both the uncorrected sensitivity analysis and the primary model. Aside from minor shifts in the sparsity thresholds at which certain effects reached significance (Supporting Information Tables S20–S22), the overall pattern of results was unchanged. The only substantive difference from the primary model was that DAN segregation shifted from correlating with executive performance to correlating with episodic performance in older adults (Supporting Information Table S21), consistent with the uncorrected sensitivity analysis (Supporting Information Table S18). Conversely, in contrast to the uncorrected sensitivity analysis but aligning with the primary results, the mediation effect of global SS on executive performance reached significance when motion was included as a covariate (Supporting Information Table S22). Finally, we note that the significant age-group effect on fMRI movement observed in the pooled sample was not present when analyzing Site 1 exclusively (younger adults: *M* = 6.01, *SD* = 5.97; older adults: *M* = 6.90, *SD* = 8.08; *t*(233) = 0.963, *p* = 0.337). Taken together, these findings indicate that neither site nor head motion substantially impacted our main results.

## DISCUSSION

This study investigated how SS relates to cognition in younger and older adults, focusing on whether its cognitive impact differs between age groups. We found that the association between global SS and semantic and executive cognition was significant in one or both age groups across a range of sparsity thresholds and involved segregation of higher-order subnetworks. These findings suggest a relationship between SS and executive cognition specific to older adults, highlighting SS as a network property supporting cognitive resilience in aging.

### Reduced System Segregation in Older Adults

These results corroborate previous findings that global SS declines with age (Chan et al., 2014; Chong et al., 2019; Pedersen et al., 2021). Specifically, we noted that older adults on average had significantly lower SS than younger adults, a result robust across sparsity thresholds despite variations that can arise from sparsity threshold choice (van den Heuvel et al., 2017; Zalesky et al., 2016). Taken together with the methodological differences of these analyses from previous work (e.g., Schaefer parcellation, binarized networks), our findings support SS decline as a robust graph-theoretic correlate of aging (Deery et al., 2023). Notably, we observed substantial within-group variability in SS, with some older adults exhibiting SS matching or exceeding younger adults, highlighting the possibility that SS can be preserved with age.

Previous cross-sectional and longitudinal studies consistently show that global SS declines with age (Chan et al., 2014; Pedersen et al., 2021), although the role of individual functional networks is less clear. Higher-order networks tend to show greater age-related reductions in segregation than primary sensory networks (Chan et al., 2014; Pedersen et al., 2021), with particularly pronounced effects in the VAN, FPN, and DMN (Chong et al., 2019). Nonetheless, within the sensory networks, prior work has found comparable desegregation of the SMN to that of association networks (Cassady et al., 2019). It remains uncertain whether these observations reflect network-specific adaptations (Kruse et al., 2023; Spreng & Turner, 2019), or vulnerability to aging (Cassady et al., 2019; Chong et al., 2017; Kong et al., 2020). Regardless, our findings reinforce prior evidence that the SMN, VAN, and FPN show particularly reduced SS in older adults.

### System Segregation Is Linked to Semantic Performance Across the Lifespan

We found that in both younger and older adults, global SS was positively related to semantic performance. At first glance, this seems inconsistent with reduced SS in older adults alongside their improved semantic abilities. However, vocabulary tends to increase with age and experience (Hartshorne & Germine, 2015; Salthouse, 2010), allowing semantic performance to improve even as the supporting functional organization declines. Supporting this view, prior work has linked higher segregation in younger adults to language production, specifically semantic retrieval (Zhang & Diaz, 2023), and to semantic intelligence more broadly (Wang et al., 2021). According to the Network Neuroscience Theory (Barbey, 2018), high functional segregation enables efficient transitions into stable, easily accessible brain states, thus facilitating semantic retrieval and performance on other relatively simple tasks (Alavash et al., 2015; Cohen & D’Esposito, 2016; Yue et al., 2017). Within this framework, the DMN is recognized beyond its established role in semantic performance (Spreng & Turner, 2019) and for its computational importance as a functional hub supporting these easily accessible state transitions (Barbey, 2018). In line with this, our results showed strong associations between DMN segregation and semantic performance in both age groups (Figure 2). Overall, our findings support a persistent association between SS and semantic performance across the lifespan, consistent with theoretical accounts of the cognitive benefits of high functional segregation.

### Age-Group Dependence of the SS-Executive Cognition Relationship

When examining executive cognition, we found that its relationship with SS was specific to older adults. This may clarify previous findings reporting a link between high SS and improved executive performance across the lifespan (Chong et al., 2019; Kong et al., 2020; Madden et al., 2020; Raykov et al., 2024), by implying that older adults may be driving these associations. In contrast, studies using exclusively younger adult samples have indicated that higher functional integration (implying lower SS) relates to improvements in executive performance (Cohen & D’Esposito, 2016; Pedersen et al., 2021; Wang et al., 2021). We observed no positive cognitive effects of lower SS, and possible reasons for this are addressed in a later section. Nonetheless, our findings indicate that analyzing age groups separately are an important consideration in understanding links between network organization and cognition.

Our finding that higher SS was linked to better executive performance only in older adults aligns with several models of network reorganization in aging. SS variance in the older group reflects not only developmental differences in network organization (Fair et al., 2007; Gu et al., 2015; He et al., 2019) but also variability in the rate of age-related decline (Chong et al., 2019; Pedersen et al., 2021). According to the compensation hypothesis, age-related dysfunction within a network prompts recruitment of other networks, reducing SS as a byproduct, thereby linking higher SS with better preserved cognition in aging (Morcom & Johnson, 2015). Alternatively, some evidence suggests that executive cognition in older adults increasingly relies on semantic memory processes, making it more dependent on the functional architecture that supports semantic cognition (Spreng & Turner, 2019), and therefore on higher segregation (Wang et al., 2021). Considering these perspectives, our findings suggest an important relationship between higher SS and preserved executive function in older adults, though further work is needed to clarify the underlying mechanisms.

### The Role of Network Parcellations in Measuring SS

A consideration in respect to these results is that SS is measured relative to predefined functional network parcellations. Rather than purely tracking how well the brain’s functional connectivity can be divided into modules, SS uniquely measures the functional organization of biologically meaningful networks, grounding the metric in systems with known cognitive roles and allowing interpretation in relation to them (Chan et al., 2014). Thus, lower global SS reflects not only reduced segregation within a participant’s networks but also differences from network organization defined by healthy young adult samples (Power et al., 2011; Schaefer et al., 2018). This detail distinguishes SS from other metrics of functional network segregation, contributing to the sensitivity and consistency of global SS in detecting age-related differences and in its positive associations with cognition (Chan et al., 2014; Deery et al., 2023; Madden et al., 2020).

However, the dependency of global SS on network parcellations may lead to inconsistencies under different parcellations, since differences in network boundaries and definitions will yield different SS despite the same underlying architecture. We used the Schaefer et al. (2018) 7-network parcellation in our analyses and observed no association between global SS and episodic memory, despite this finding being replicable under the Power et al. (2011) parcellation (Chan et al., 2014; Kong et al., 2020). In a single sample, Pedersen et al. (2021) found SS correlated with episodic memory when using the Power parcellation but not the Schaefer parcellation. Importantly, the Power memory subnetwork overlaps substantially with the Schaefer DMN and FPN, such that strong segregation of this subnetwork could appear as reduced segregation of the DMN and FPN, potentially explaining their negative trend with episodic cognition observed here in younger adults. In contrast, the DMN and FPN, as especially implicated in semantic cognition (Spreng & Turner, 2019), and the broader set of higher-order networks involved in executive cognition (Menon & D’Esposito, 2021), are more consistently included across parcellations (Bryce et al., 2021). However, even within analogous networks, variation in parcellation boundaries can systematically affect network measures and their correlations with individual differences in cognition (Bryce et al., 2021), and our findings support this also applies to SS (Pedersen et al., 2021). While a full cross-parcellation analysis was beyond this study’s scope, our results demonstrate associations under the Schaefer 7-network parcellation between age, SS, and executive and semantic cognition.

### Limitations

The primary limitation of our methodology was its cross-sectional design, which prevented us from determining whether low SS reflected age-related decline or stable individual differences. In addition, this analysis used multisite data, providing larger and more diverse samples that improve generalizability and were important for detecting the small cognitive effects observed in SS research (Chan et al., 2014; Pedersen et al., 2021), comparable to those observed here. However, the racial and ethnic diversity of the sample remained limited, particularly among older adults, which reduced the generalizability of our findings and may have introduced bias. Furthermore, we observed significant site-related differences in demographic, cognitive, and network variables, as well as in fMRI movement. This made it challenging to separate natural demographic variation from methodological site differences, particularly given the known impact of site effects and head motion on functional connectivity estimates (Noble et al., 2017; Power et al., 2012). However, after accounting for site and fMRI movement in a sensitivity analysis, the main findings remained robust: SS was reduced in older adults, and lower SS was associated with poorer executive cognition in older adults and weaker semantic performance across the lifespan.

## CONCLUSIONS

Our findings suggest that the cognition of older adults may be more sensitive to variance in SS than younger adults, or that declining SS is especially detrimental to executive cognition. Thus, coupled with emerging evidence that higher SS is protective against age-related diseases such as dementia (Chan et al., 2021; Ewers et al., 2021; Zhang et al., 2023), the maintenance of system segregation is implicated as an important target in supporting cognitive health in aging. Although the factors that promote SS maintenance with aging remain unclear (Raykov et al., 2024), some evidence suggests that exercise may help preserve SS (Kommula et al., 2024). Altogether, these findings highlight SS as a key metric for further investigation in understanding how brain network organization relates to cognitive aging.

## Supporting Information

Supporting information for this article is available at https://doi.org/10.1162/NETN.a.542.

## Author Contributions

Cameron Nowlan Calder: Conceptualization; Formal analysis; Methodology; Software; Validation; Visualization; Writing – original draft; Writing – review & editing. Carl Helmick: Data curation; Resources; Software. Javeria Ali Hashmi: Conceptualization; Formal analysis; Funding acquisition; Methodology; Project administration; Software; Supervision; Writing – review & editing.

## Funding Information

We would like to acknowledge our funding sources: Javeria Ali Hashmi’s work was supported by The Canada Research Chairs Program, the John R. Evans Leaders and Canada Innovation Funds (CFI-JELF), the Canadian Institute of Health Research (CIHR) Project Grant. Cameron Calder’s work was supported by the Brain Repair Centre through the Dalhousie Faculty of Medicine 2025 Graduate Studentship program and by the CIHR through the Canada Graduate Scholarship - Master’s (CGS-M).

## Supplementary Material



## TECHNICAL TERMS

System segregation: A measure comparing the strength of connectivity within the same functional brain network to connectivity between different functional brain networks.
Segregation: A graph theoretic concept describing the tendency for nodes to form tightly connected groups with limited connections to other groups.
Parcellation: Partitioning of the brain into regions of interest (ROIs) and/or subnetworks, based on anatomical and functional characteristics.
Binarized (system segregation): Setting all functional connections surviving sparsity thresholding to 1, making the network unweighted and thereby emphasizing architecture over connection strength.
Homogeneity of variance (or homoscedasticity): The assumption that compared groups have relatively equal variance; violations can undermine statistical test validity.
Sparsity threshold: A cutoff value used to retain a distinct subset of the possible connections in a brain network, creating a distinct network structure.
Higher-order (network): Brain networks supporting complex cognition, incorporating multiple sensory-motor modalities (DAN, VAN, limbic network, FPN, DMN in Schaefer-7 parcellation).
Primary (network): Brain networks for basic sensory-motor functions (visual and somatomotor networks in Schaefer-7 parcellation).
Robust regression: Regression method that reduces the weights of outliers to give more reliable estimates.
Partial correlation: Test of association between two variables while controlling for one or more covariates.
Range-restriction correction: A statistical adjustment used to correct for reduced variance in a sample that may obscure true associations.
