# Supplementary material for: High brain network system segregation is differentially linked with cognitive performance across the life span
Source: Netw Neurosci. 2026 Apr 22;10(2):352–73. doi: 10.1162/NETN.a.542 (PMC13108503; doi:10.1162/NETN.a.542)
Supplement: Supplementary file 1 [file netn-10-2-352-s001.pdf]

Supplementary material

Normality and Variance of System Segregation (SS)........ 1
Levene's test (**Table S1**) 1
Shapiro-Wilk test (**Table S2**) 2
Distribution of key variables (**Figure S1**) 3
Distribution of subnetwork SS (**Figure S2**) 4
Threshold-Specific Analysis Reports........ 5
Age-group differences in SS (**Table S3**) 5
SS and cognition in younger adults (**Table S4**) 6
SS and cognition in older adults (**Table S5**) 7
Additional Analyses........ 8
Robust regression (**Table S6**) 8
Effect of within-group continuous age (**Figure S3**) 9
Age-group prediction with logistic regression (**Table S7**) 10
Age-group prediction with random forest (**Table S8**) 11
Mediation effect on episodic cognition (**Figure S4**) 12
Sex effects on SS and Cognition 13
Effects of sex on SS (**Table S9**) 13
Effects of sex on cognition (**Table S10**) 14
Sex-Unadjusted Analyses (**Tables S11-S13**) 15
Sensitivity of Results to Site Effects 18
Site differences in demographics (**Table S14**) 18
Site differences in SS metrics (**Table S15**) 19
Site differences in cognition (**Table S16**) 20
Sensitivity analyses excluding site 2 (**Tables S17-19**) 24
Motion Corrected Analyses 18
Age-group differences in SS, motion corrected (**Table S20**) 18
SS and cognition, motion corrected (**Table S21**) 19
Mediation analysis, motion corrected (**Table S22**) 20

Normality and Variance of System Segregation

Network	Sparsity Threshold (T)	Levene's test (*F*)	Sig. (*p*)	Greater variance
Global	.05–.45	3.931 to 7.925	.005–.048	Older
Visual	.05–.30	4.158 to 21.385	< .001–.042	Older
SMN	.10–.45	7.370 to 14.658	< .001–.007	Older
DAN	.05	4.921	.027	Older
VAN	.05–.50	4.868 to 18.615	< .001–.028	Older
Limbic	.10–.20	4.452 to 8.842	.003–.036	Older
DMN	.10–.15	4.200 to 5.452	.020–.041	Older

Table S1. Levene's test for equality of variance in SS between younger (n = 179) and older age groups (n = 117). Significant results are reported for thresholds at which the assumption of equal variance was violated. Variance was consistently greater in older adults, with violations observed at 9 out of 10 thresholds for global segregation and 31 out of 70 thresholds across functional sub-networks.

Age-group	Network	Sig. Sparsity Thresholds	Shapiro-Wilk (*W*)	Sig. (*p*)
Younger	Global	.05–.45	0.950 to 0.981	< .001–.015
	Visual	.05–.35	0.922 to 0.981	< .001–.014
	SMN	.05–.35	0.943 to 0.980	< .001–.012
	DAN	.05–.45	0.959 to 0.982	< .001–.022
	VAN	.05–.20, .30–.45	0.908 to 0.984	< .001–.044
	Limbic	.05–.15	0.827 to 0.963	< .001
	FPN	.05–.50	0.933 to 0.978	< .001–.006
	DMN	.05–.50	0.956 to 0.981	< .001
Older	Global	.05–.50	0.901 to 0.970	< .001–.010
	Visual	.05–.35	0.714 to 0.971	< .001–.013
	SMN	.05–.50	0.835 to 0.923	< .001
	DAN	.05–.45	0.846 to 0.971	< .001–.011
	VAN	.05–.50	0.864 to 0.953	< .001
	Limbic	.05–.30	0.859 to 0.974	< .001–.023
	FPN	.05–.50	0.924 to 0.967	< .001–.006
	DMN	.05–.50	0.890 to 0.969	< .001–.009

Table S2. Shapiro-Wilk test for normality of SS distributions in younger (n = 179) and older (n = 117) age groups. Significant violations of normality are shown across networks, sparsity thresholds and age groups. Deviations from normality were observed in all networks for both age groups, with more overall violations observed in the older age group (73/80 thresholded networks) compared to the younger group (63/80 thresholded networks).

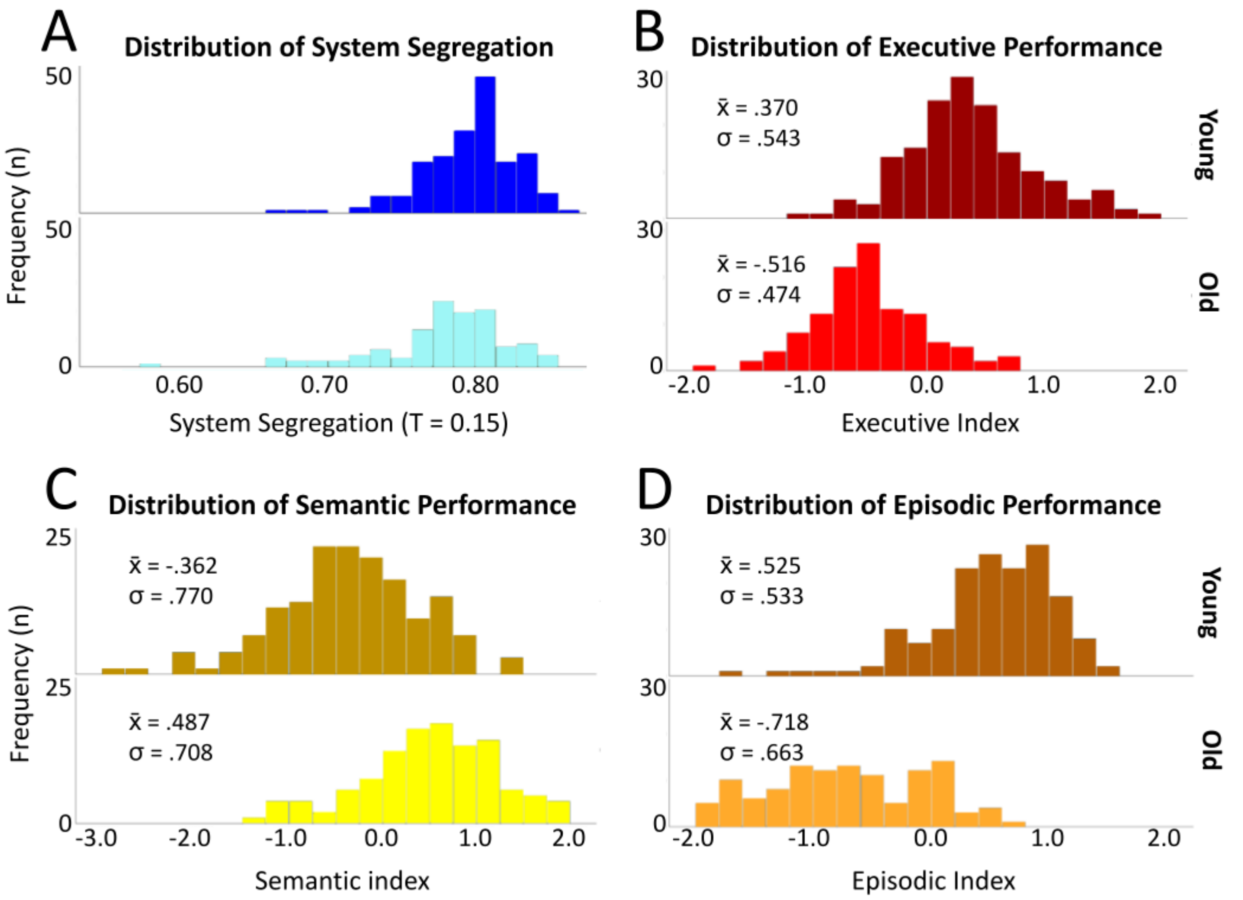

Figure S1. Distributions of our investigated cognitive domains and SS (T = 0.15) in older and young age groups. A) Variance in SS was higher in older (n = 117) compared to younger (n = 179) adults (in Table S1) and was non-normally distributed in both groups (in Table S2). B) Variance in executive performance was higher in young adults (n = 161) compared to older adults (n = 117), but this effect was not significant (F = 1.405, p = 0.237). The Shapiro-Wilk test revealed executive performance violated normality in the young group (W = .982, p = .032) but not the older group (W = .989, p = .460). C) Variance in semantic performance was higher in young adults compared to older adults, but this effect was not significant (F = 0.321, p = 0.572). The Shapiro-Wilk test revealed normal distributions of executive performance in the young (W = .987, p = .135) and older group (W = .982, p = .124). D) Variance in episodic performance was significantly higher in older adults compared to young adults as detected by Levene's test for equality of variance (F = 12.785, p < 0.001). The Shapiro-Wilk test revealed that episodic performance violated normality in both the young (W = .943, p < .001) and older groups (W = .975, p = .029). Mean value (x̄) and variance (σ) were reported for each distribution.

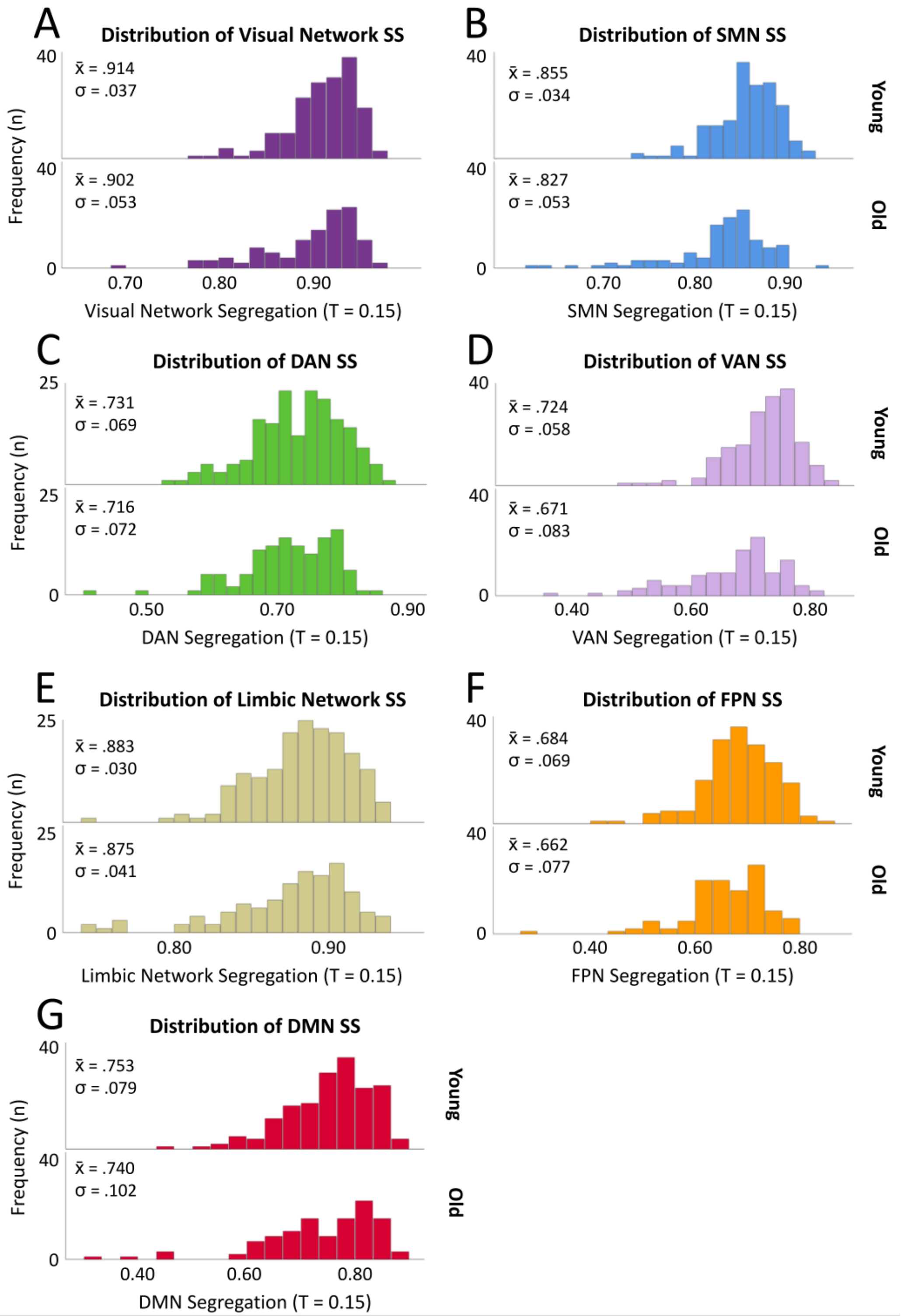

Figure S2. Distributions of subnetwork SS in younger and older adults. We used SS at a sparsity threshold (T) of 0.15 to visualize these results. See Table S1 for Levene's test of non-homogeneity of variance in subnetwork SS between younger and older adults, and Table S2 for testing of normality of variance using the Shapiro-Wilke test. Mean value ($\bar{x}$) and variance (σ) were reported for each distribution.

Threshold-Specific Analysis Reports

Network	T	*F(1,294)*	Sig. (*p*)	Higher segregation
Global	.05	7.25	.008	Younger
	.10	16.76	< .001	Younger
	.15	16.65	< .001	Younger
	.20	13.83	< .001	Younger
	.25	9.36	.002	Younger
	.30	6.60	.011	Younger
	.35	4.67	.031	Younger
	.50	6.25	.013	Younger
SMN	.05	6.08	.014	Younger
	.10	21.48	< .001	Younger
	.15	28.99	< .001	Younger
	.20	29.81	< .001	Younger
	.25	19.78	< .001	Younger
	.30	13.12	< .001	Younger
	.35	8.64	.004	Younger
	.40	5.40	.021	Younger
	.50	4.99	.026	Younger
VAN	.05	23.04	< .001	Younger
	.10	35.28	< .001	Younger
	.15	36.00	< .001	Younger
	.20	38.40	< .001	Younger
	.25	37.89	< .001	Younger
	.30	38.06	< .001	Younger
	.35	38.82	< .001	Younger
	.40	40.05	< .001	Younger
	.45	38.75	< .001	Younger
	.50	53.42	< .001	Younger
FPN	.10	4.53	.034	Younger
	.15	5.32	.022	Younger
	.20	6.55	.011	Younger
	.25	7.31	.007	Younger
	.30	10.78	.001	Younger
	.35	10.85	.001	Younger
	.40	10.62	.001	Younger
	.45	9.30	.002	Younger
	.50	12.93	< .001	Younger

Table S3. Age-group differences in SS. Reported as in Table 2 of the manuscript, but with statistics reported for each threshold (T) rather than as a range across significant thresholds.

Cognitive Domain	Network	T	Correlation (r_s)	Sig. (p)
Executive	DAN	.20	.162	.041
		.25	.163	.040
		.35	.162	.042
		.40	.165	.038
		.45	.159	.046
Semantic	Global	.05	.192	.015
		.10	.173	.029
		.15	.199	.012
		.20	.170	.032
		.25	.162	.042
		.35	.159	.045
	DAN	.20	.165	.038
		.25	.177	.025
		.30	.165	.038
		.35	.172	.030
		.40	.161	.043
	DMN	.05	.173	.029
		.40	.157	.048
		.45	.157	.048

Table S4. Relationship between SS and cognition in younger adults. Reported as in Table 3 of the manuscript, but with statistics reported for each threshold (T) rather than as a range across significant thresholds.

Cognitive Domain	Network	T	Correlation (r_s)	Sig. (*p*)
Executive	Global	.05	.207	.027
		.10	.237	.011
		.15	.237	.011
		.20	.220	.018
		.25	.205	.028
		.30	.199	.033
	DAN	.05	.228	.014
		.10	.241	.009
		.15	.204	.028
		.20	.204	.029
		.30	.196	.036
	VAN	.05	.213	.022
		.10	.240	.010
		.15	.253	.006
		.20	.206	.027
	Limbic	.05	.285	.002
		.10	.284	.002
		.15	.244	.009
	DMN	.05	.319	< .001
		.10	.302	.001
		.15	.280	.002
		.20	.265	.004
		.25	.247	.008
		.30	.247	.008
		.35	.231	.013
		.40	.214	.022
		.45	.213	.022
		.50	.201	.032
Semantic	Global	.05	.201	.031
		.10	.228	.014
		.15	.203	.030
	VAN	.05	.185	.048
		.10	.209	.025
		.20	.197	.035
	FPN	.05	.241	.009
		.10	.224	.016
		.20	.228	.014
		.25	.206	.027
	DMN	.05	.209	.025
		.10	.213	.022
		.15	.185	.048

Table S5. Relationship between SS and cognition in younger adults. Reported as in Table 3 of the manuscript, but with statistics reported for each threshold (T) rather than as a range across thresholds.

Additional Analyses

Network	Sig. Sparsity Thresholds	Effect size (*β*)	Sig. (*p*)	Higher segregation
Global	.05–.30, .50	-0.007 to -0.013	.001–.024	Younger
SMN	.05–.30	-0.007 to -0.019	< .001–.025	Younger
VAN	.05–.50	-0.033 to -0.043	< .001	Younger
FPN	.10–.50	-0.010 to -0.031	< .001–.040	Younger

Table S6. Robust regression to detect age-group differences in SS. Utilizing a robust regression, with age-group set as a binary variable, allowed us to confirm age-group differences with less sensitivity to outliers. Robust regression was run using the robustfit function in MATLAB with default parameters.

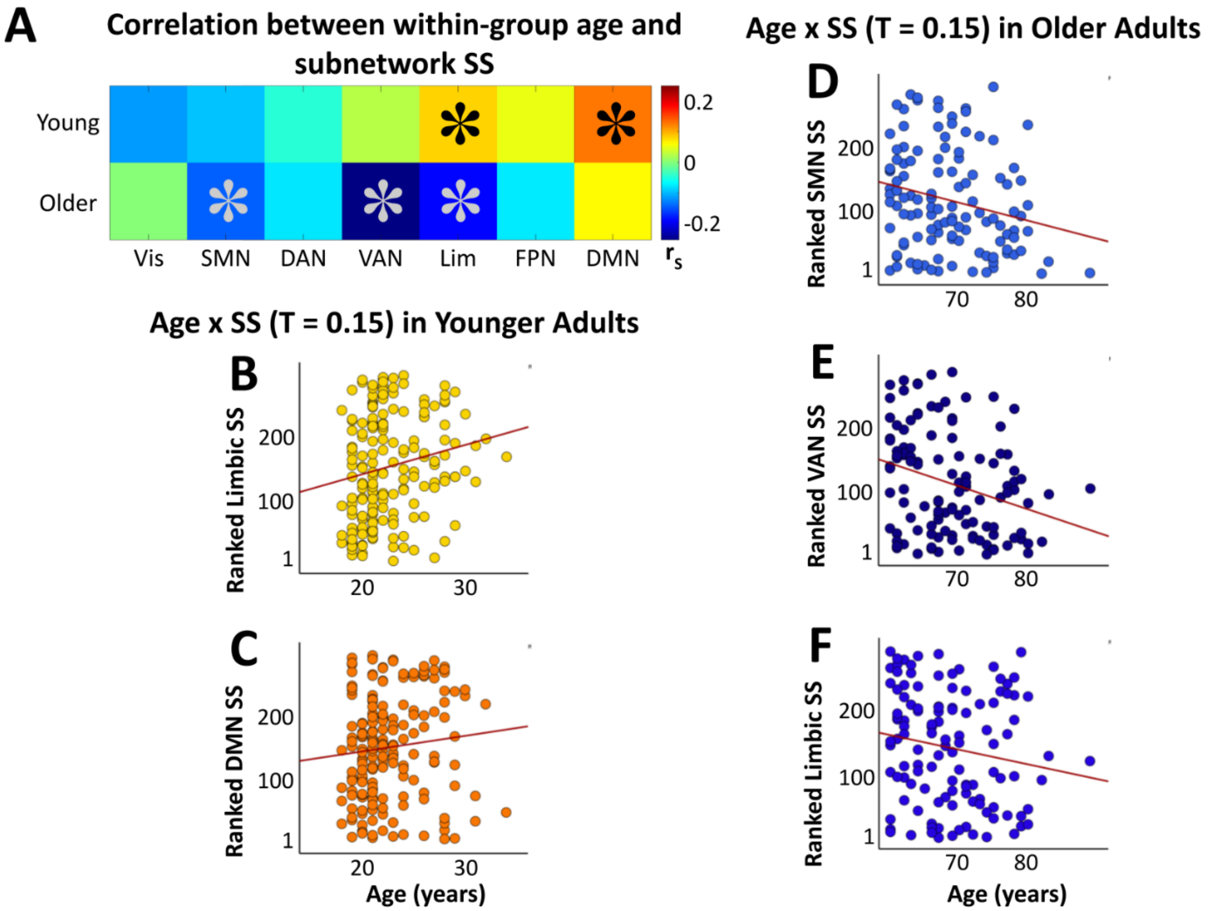

Figure S3. The relationship between within-group age on SS and cognition. A) Spearman correlation (r_s) between subnetwork level segregation and within-group age in young and older adults. * indicates significance at the 0.05 level across 3 or more sparsity thresholds. In the younger group, significant correlations were found for within-group age with the limbic network ($T^{0.05\text{-}0.20}$: r_s = 0.259, p < 0.001; r_s = 0.230, p = 0.002; r_s = 0.224, p = 0.003; r_s = 0.158, p = 0.036) and the DMN ($T^{0.05\text{-}0.20}$: r_s = 0.179, p = 0.017; r_s = 0.163, p = 0.030; r_s = 0.148, p = 0.049). In the older group, significant correlations were found between within-group age and the SMN ($T^{0.05\text{-}0.20}$: r_s = -0.278, p = 0.002; r_s = -0.268, p = 0.004; r_s = -0.222, p = 0.017; r_s = -0.186, p = 0.045) the VAN ($T^{0.05\text{-}0.45}$: r_s = -0.270, p = 0.003; r_s = -0.292, p = 0.001; r_s = -0.315, p < 0.001; r_s = -0.283, p = 0.002; r_s = -0.284, p = 0.002; r_s = -0.274, p = 0.003; r_s = -0.245, p = 0.008; r_s = -0.221, p = 0.017; r_s = -0.191, p = 0.040) and the limbic network ($T^{0.20\text{-}0.45}$: r_s = -0.246, p = 0.008; r_s = -0.249, p = 0.007; r_s = -0.273, p = 0.003; r_s = -0.287, p = 0.002; r_s = -0.267, p = 0.004; r_s = -0.208, p = 0.025). Scatterplots (Figures B–F) display the relationships between within-group age (x-axis) and rank-ordered subnetwork SS at T = 0.15 (y-axis). We note here that for global SS, significance was not found for 3 or more sparsity thresholds, and for cognition, significant correlations were found only in the older group, with executive (r_s = -0.247, p = 0.007) semantic (r_s = 0.239, p = 0.010) and episodic performance (r_s = -0.346, p < 0.001).

Model	Metric	Mean difference (B-A)	Statistical difference (*t*)	Sig. (*p*)
Logistic Regression	AUC	0.0018	0.7140	.5147
	bal. ACC	0.0024	0.2901	.7862
	ACC	0.0045	0.5345	.6213
Random Forest	AUC	-0.0051	-0.6706	.5392
	bal. ACC	-0.0044	-0.2291	.8300
	ACC	0.0002	0.0117	.9912

Table S7. Statistical comparisons of training performance between cognition-only (episodic, semantic, and executive memory) and cognition+SS (cognition plus SS across 10 sparsity thresholds) feature sets. Paired t-tests were conducted across identical training folds using both logistic regression and random forest classifiers. Reported metrics include area under the curve (AUC), accuracy (ACC), and balanced accuracy (Bal. ACC).

	Training Performance (avg)			Hold-out Test Performance		
**Model Features**	**AUC**	**ACC**	**Bal. ACC**	**AUC**	**ACC**	**Bal. ACC**
Cognition	0.977	0.914	0.911	1.000	0.982	0.979
Cognition+SS	0.978	0.914	0.913	0.999	0.984	0.982
Cognition+SS+Sex	0.986	0.941	0.937	0.987	0.927	0.929

Table S8. Training and hold-out test (70/30 split) performance of logistic regression models predicting age group. Feature sets included: cognition-only (episodic, semantic, executive memory), cognition+SS (cognition plus SS across 10 sparsity thresholds), and cognition+SS+sex (adding sex as a binary variable). Metrics reported are area under the curve (AUC), accuracy (ACC), and balanced accuracy (Bal. ACC).

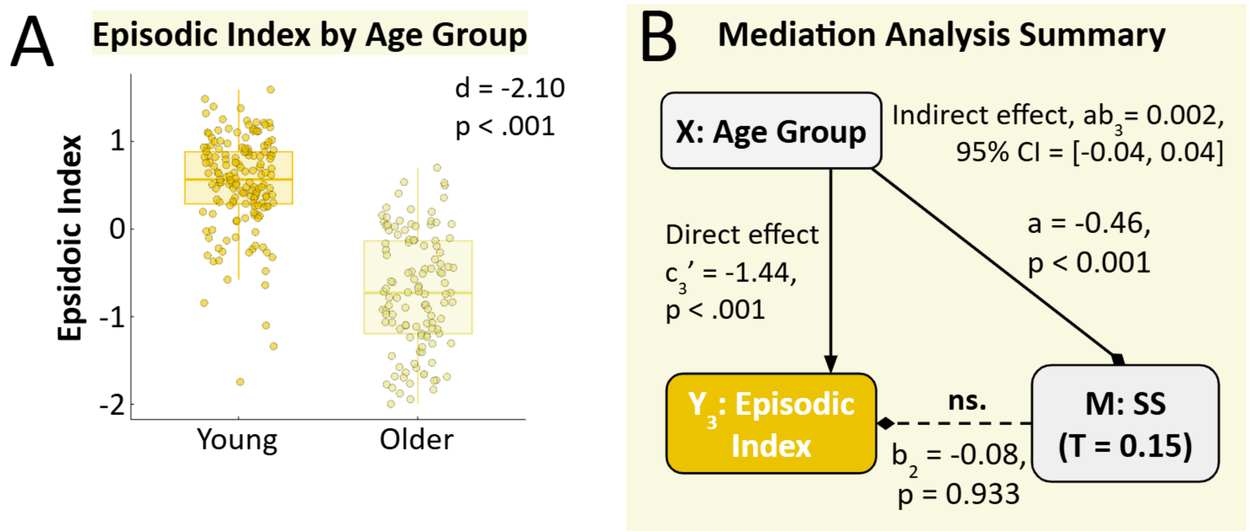

Figure S4. SS does not significantly mediate an effect of age-group on the episodic index. A) Univariate scatter plot displaying group differences in the episodic index between young (18-29 years) and older (60-89 years) adults. B) Diagram showing our full PROCESS mediation analysis on the effect of age group on the episodic index with SS (T = 0.15) as the mediator. Group-relative age and sex were included as covariates. The arrows show predictive strength of X: age group and M: SS in regression models, with arrow end indicating a negative effect, ➤ indicating a positive effect, and the dashed line from SS to Episodic index emphasizing the non-significance of the effect. The confidence interval for the overall indirect effect was derived at a confidence level of 95% using 5000 bootstrap samples.

Sex Effects on SS and Cognition

Network	Sig. Sparsity Thresholds	*F(1,294)*	Sig. (*p*)	Higher segregation
**Global Segregation**				
Total	.10–.50	5.06 to 7.56	.006–.025	Female
Younger group	ns.	–	–	–
Older group	.10–.20	4.21 to 5.27	.024–.043	Female
**DAN Segregation**				
Total	.10–.40	4.07 to 5.20	.023–.045	Female
Younger group	ns.	–	–	–
Older group	ns.	–	–	–
**VAN Segregation**				
Total	.05–.25	4.19 to 11.13	< .001–.049	Female
Younger group	ns.	–	–	–
Older group	.05–.15	4.74 to 7.42	.007–.032	Female
**DMN Segregation**				
Total	.05–.50	7.56 to 10.73	.001–.006	Female
Younger group	.05–.50	5.34 to 7.50	.007–.022	Female
Older group	.10, .20–.50	4.01 to 4.67	.033–.048	Female

Table S9. Effects of sex on global and subnetwork SS metrics. The statistics reported here were calculated using rank-ordered two-way ANOVAs, with age group and sex set as the independent variables, and group age included as a covariate. Reported ranges include only values from significant sparsity thresholds; *ns.* indicates effects not significant at three or more thresholds.

Cognitive Metric	Male	Female	*F(1,275)*	Sig. (*p*)	Higher group
**Executive Index** *M* (*SD*)					
Total	-0.034 (0.695)	0.022 (0.661)	0.33	0.556	–
Younger group	0.374 (0.537)	0.367 (0.549)	0.02	0.877	–
Older group	-0.560 (0.493)	-0.479 (0.459)	1.28	0.261	–
**Semantic Index** *M* (*SD*)					
Total	0.083 (0.757)	-0.072 (0.918)	1.35	0.246	–
Younger group	-0.207 (0.684)	-0.475 (0.812)	5.01	0.027*	Male
Older group	0.456 (0.684)	0.512 (0.732)	0.06	0.801	–
**Episodic Index** *M* (*SD*)					
Total	-0.208 (0.893)	0.163 (0.785)	26.12	< 0.001*	Female
Younger group	0.369 (0.592)	0.639 (0.457)	9.61	0.002*	Female
Older group	-0.948 (0.628)	-0.527 (0.635)	16.54	< 0.001*	Female

Table S10. Effects of sex on cognitive performance. The statistics reported here were calculated using rank-ordered two-way ANOVAs, with age group and sex set as the independent variables, and group age included as a covariate.

Network	Sig. Sparsity Thresholds	*F(1,294)*	Sig. (*p*)	Higher Segregation
Global	.05–.40, .50	4.14–16.70	< .001–.043	Younger
SMN	.05–.40, .50	5.96–30.17	< .001–.015	Younger
VAN	.05–.50	22.97–55.08	< .001 for all	Younger
FPN	.10–.50	4.60–13.87	< .001–.033	Younger

Table S11. Sex unadjusted differences in SS between younger and older adults. The statistics reported here were calculated using rank-ordered one-way ANOVAs, with age group set as the independent variable. Ranges of statistics reported here include only values from the indicated significant sparsity thresholds.

Age group	Cognitive Domain	Network	Sig. Sparsity Thresholds	Correlation (r_s)	Sig. (*p*)
Younger	Executive	DAN	.35–.50	.161–.175	.027 to .042
	Episodic	DAN	.20, .25 .35–.45	.159–.165	.037 to .044
Older	Executive	Global	.05–.35	.184–.254	.006 to .048
		DAN	.05–.35	.198–.258	.005 to .033
		VAN	.05–.25	.190–.269	.003 to .041
		Limbic	.05–.15	.219–.257	.005 to .018
		DMN	.05–.50	.218–.331	<.001 to .019
	Semantic	Global	.05–.15	.204–.230	.013 to .028
		VAN	.05, 0.10, .20	.187–.211	.023 to .044
		FPN	.05–.10, .20–.30	.188–.234	.012 to .044
		DMN	.05–.15	.188–.216	.020 to .044
	Episodic	DAN	.10–.40	.195–.232	.012 to .036
		VAN	.15–.25	.191–.218	.019 to .040

Table S12. Sex-unadjusted relationship between SS and cognition in younger and older adults. Analyses were corrected for within-group age using partial correlations. Reported ranges of r_s values and p-values reflect only those sparsity thresholds showing significant (α = .05) correlations with the indicated cognitive index.

Mediation step	Sig. Sparsity Thresholds	Standardized effect (*B*)	Sig.
Age group → SS (*a*)	.05–.50	-0.268 to -0.471	p < .001 to p = .048
SS → executive performance (b_1)	.05–.15	0.096 to 0.108	p = .012 to .040
Age group → SS → executive performance (ab_1)	.05–.15	-0.027 to -0.034	95% CI = [-0.059, -0.004] to [-0.072, -0.005]
SS → semantic performance (b_2)	.05–.20	0.113 to 0.144	p = .008 to .035
Age group → SS → semantic performance (ab_2)	.05–.20	-0.041 to -0.058	95% CI = [-0.089, -0.006] to [-0.113, -0.016]

Table S13. Sex-unadjusted PROCESS mediation model. Sex and group-relative age was included as a covariate. Results are reported as ranges across all significant sparsity thresholds (α = .05) or as 95% bootstrap confidence intervals (5,000 samples), which did not include zero. The mediation effect of SS on episodic performance was significant at no sparsity thresholds.

Sensitivity of Results to Site Effects on SS and Cognition

	Ithaca (Site 1)	Toronto (Site 2)	Group difference	Sig. (*p*)
**Age group** (% older group)	35.3%	55.7%	$\chi^2 = 8.45$	.004
**Ethnicity** (% Hispanic)	7.7%	1.6%	$\chi^2 = 2.24$	.134
**Race**				
White	72.8%	60.7%	$\chi^2 = 4.26$	.039
Asian	11.1%	13.1%	$\chi^2 = 0.22$	.642
Black	5.5%	8.2%	$\chi^2 = 0.62$	.430
Other	2.1%	8.2%	$\chi^2 = 5.53$	.019
Not provided	9.4%	9.8%	–	–
**Sex** (% female)				
Total	56.6%	55.7%	$\chi^2 = 0.02$	.904
Younger group	56.6%	63.0%	$\chi^2 = 0.38$	.536
Older group	56.6%	50.0%	$\chi^2 = 0.43$	.513
**Age** *M* (*SD*)				
Total	38.31 (22.10)	50.49 (24.34)	U = 4617	< .001
Younger group	22.31 (3.14)	24.26 (3.62)	t = -2.91	.004
Older group	67.60 (5.70)	71.32 (7.54)	U = 1002	.014
**fMRI Movement** *M* (*SD*)				
Total	6.32 (6.79)	21.31 (13.85)	U = 1918	< .001
Younger group	6.01 (5.97)	18.94 (12.13)	U = 575	< .001
Older group	6.90 (8.08)	23.19 (14.98)	U = 363	< .001
**Years of Education** *M* (*SD*)				
Total	16.18 (2.69)	15.61 (2.12)	U = 6126	.269
Younger group	15.28 (1.99)	14.81 (1.50)	t = 1.16	.248
Older group	17.67 (3.03)	16.24 (2.35)	t = 2.48	.015

Table S14. Demographic differences between site 1 (Ithaca) and site 2 (Toronto). Chi-square tests were used to assess age-group differences in categorical variables, and independent t-tests or Mann–Whitney U tests were used for continuous variables, depending on whether Levene's test indicated a violation of the homogeneity of variance assumption.

	Ithaca *M* (*SD*)	Toronto *M* (*SD*)	Sig. Sparsity Thresholds	Group difference	Sig. (*p*)
**Global Segregation**					
Total	0.67 (0.04)	0.67 (0.04)	ns.	–	–
Younger group	0.68 (0.03)	0.68 (0.03)	ns.	–	–
Older group	0.66 (0.04)	0.66 (0.04)	ns.	–	–
**Visual Segregation**					
Total	0.80 (0.06)	0.76 (0.06)	.10–.50	3840–5359 (*U*)	< .001–.002
Younger group	0.80 (0.05)	0.77 (0.06)	.25–.50	1117–1486 (*U*)	< .001–.023
Older group	0.80 (0.07)	0.76 (0.06)	.05–.50	771–1030 (*U*)	< .001–.022
**SMN Segregation**					
Total	0.73 (0.04)	0.71 (0.06)	.05–.20, .50	5716–5994 (*U*)	.049–.015
Younger group	0.73 (0.03)	0.72 (0.04)	ns.	–	–
Older group	0.71 (0.05)	0.70 (0.07)	ns.	–	–
**DAN Segregation**					
Total	0.62 (0.07)	0.63 (0.06)	ns.	–	–
Younger group	0.63 (0.06)	0.63 (0.07)	ns.	–	–
Older group	0.61 (0.07)	0.63 (0.05)	ns.	–	–
**VAN Segregation**					
Total	0.60 (0.06)	0.59 (0.06)	ns.	–	–
Younger group	0.61 (0.05)	0.62 (0.04)	ns.	–	–
Older group	0.57 (0.07)	0.56 (0.07)	ns.	–	–
**Limbic Segregation**					
Total	0.74 (0.04)	0.75 (0.05)	ns.	–	–
Younger group	0.75 (0.04)	0.75 (0.05)	ns.	–	–
Older group	0.74 (0.05)	0.75 (0.05)	ns.	–	–
**FPN Segregation**					
Total	0.58 (0.07)	0.58 (0.07)	ns.	–	–
Younger group	0.59 (0.07)	0.60 (0.07)	ns.	–	–
Older group	0.57 (0.07)	0.57 (0.07)	ns.	–	–
**DMN Segregation**					
Total	0.63 (0.09)	0.68 (0.08)	.05–.50	-3.58 to -4.18 (*t*)	< .001
Younger group	0.64 (0.08)	0.70 (0.07)	.05–.50	-3.03 to -3.80 (*t*)	< .001–.003
Older group	0.62 (0.10)	0.67 (0.09)	.05–.50	-2.01 to -2.51 (*t*)	.013–.038

Table S15. Differences in global and subnetwork SS metrics between site 1 (Ithaca) and site 2 (Toronto). Group differences were tested and reported with t-tests, or with Mann–Whitney U tests in cases where Levene's test indicated non-homogeneity of variance across 3 or more thresholds. If sparsity thresholds did not show significant group differences across 3 or more thresholds, they were not reported (ns.).

	Ithaca (Site 1)	Toronto (Site 2)	Group difference	Sig. (*p*)
**Executive Index** *M* (*SD)*				
Total	-0.02 (0.68)	0.05 (0.66)	t = -0.62	.534
Younger group	0.35 (0.52)	0.45 (0.66)	U = 1615	.380
Older group	-0.61 (0.45)	-0.28 (0.45)	t = -3.86	< .001
**Semantic Index** *M* (*SD)*				
Total	0.02 (0.82)	-0.08 (0.98)	t = 0.79	.431
Younger group	-0.28 (0.73)	-0.76 (0.85)	t = 2.98	.003
Older group	0.50 (0.71)	0.45 (0.71)	t = 0.32	.752
**Episodic Index** *M* (*SD)*				
Total	0.12 (0.81)	-0.42 (0.89)	t = 4.55	< .001
Younger group	0.58 (0.47)	0.25 (0.73)	U = 1386	.056
Older group	-0.62 (0.67)	-0.95 (0.58)	t = 2.55	.012

Table S16. Cognitive performance differences between site 1 (Ithaca) and site 2 (Toronto). Group differences were tested and reported with t-tests, or with Mann–Whitney U tests in cases where Levene's test indicated non-homogeneity of variance.

Network	Sig. Sparsity Threshold (T)	*F(1,294)*	Sig. (*p*)	Higher segregation
Global	.05–.30	4.19 to 12.67	< .001–.042	Younger
SMN	.05–.35	6.53 to 27.57	< .001–.011	Younger
VAN	.05–.50	14.45 to 38.14	< .001 for all	Younger
FPN	.30–.50	5.31 to 8.63	.004–.022	Younger

Table S17. Differences in SS between younger (n = 152) and older (n = 83) adults with site 2 excluded. The statistics reported here were calculated using rank-ordered two-way ANOVAs, with age group and sex set as the independent variables. Ranges of statistics reported here include only values from the indicated significant sparsity thresholds.

Age-group	Cognitive Domain	Network	Sig. Sparsity Thresholds	Correlation (r_s)	Sig. (*p*)
Younger	Semantic	Global	.05–.50	.195 to .272	.002–.025
Younger	Semantic	DAN	.10–.50	.175 to .238	.006–.045
Younger	Semantic	DMN	.05–.50	.258 to .325	< .001–.003
Older	Executive	Global	.10–.35	.228 to .269	.015–.040
Older	Executive	VAN	.10–.20	.220 to .255	.022–.049
Older	Executive	Limbic	.10–.20	.249 to .298	.007–.025
Older	Executive	DMN	.05–.20	.220 to .303	.006–.048
Older	Semantic	Global	.05–.35	.222 to .318	.004–.047
Older	Semantic	VAN	.05–.20	.229 to .296	.007–.039
Older	Semantic	FPN	.05, .10, .20	.222 to .249	.025–.047
Older	Semantic	DMN	.05–.20	.246 to .294	.008–.018
Older	Episodic	DAN	.05–.45	.224 to .277	.012–.045

Table S18. Cognition remains correlated with global SS and subnetwork segregation following the restriction of the analysis to site 1 (Ithaca; n = 217). Results are reported as a range of values across all significant (α = 0.05) sparsity thresholds for significant, age and sex controlled, spearman correlations (r_s) that showed significance for at least three sparsity thresholds. In younger adults (n = 134), semantic cognition was correlated with segregation measures, while executive and episodic cognition were not. Replicating our primary analysis, the effect of segregation was much more widespread in the older group (n = 83), with significant correlations observed for semantic, episodic and executive cognition.

Mediation analysis step	Sparsity Threshold (T)	Effect size (*B*)	Sig.
Age-group → SS (a)	.05–.30, .50	-0.307 to -0.485	p < .001 to p = .028
SS → executive performance (b)	.05–.10	0.106	p = .034 to .035
Age-group → SS → executive performance (ab)	.05–.15	-0.043 to -0.051	95% CI = [-0.101, -0.003] to [-0.108, -0.008]
SS → semantic performance (b)	.05–.30, .50	0.126 to 0.234	p < .001 to p = .039
Age-group → SS → semantic performance (ab)	.05–.35	-0.046 to -0.114	95% CI = [-0.111, -0.002] to [-0.201, -0.043]

Table S19. Global SS mediates a negative effect of age-group on cognition. Results are reported as a range of values across all significant (α = 0.05) sparsity thresholds in the PROCESS mediation model. Age Group was a binary predictor set as either young or older; sex and group-relative age were set as covariates. Confidence intervals were derived at a confidence level of 95% using 5000 bootstrap samples. When restricting the primary analysis to site 1 (Ithaca; n = 217), the mediation effect of SS on executive performance was lost while the effect on semantic performance became more robust. The total effect of Age-group on semantic performance was positive (B = 0.8690, p < 0.001 in a model with T0.15).

Network	Sig. Sparsity Threshold (T)	*F(1,294)*	Sig. (*p*)	Higher segregation
Global	.05–.25	5.26 to 11.79	< .001–.023	Younger
SMN	.05–.35	6.22 to 26.90	< .001–.013	Younger
VAN	.05–.50	13.55 to 37.44	< .001 for all	Younger
FPN	.30–.50	5.05 to 8.17	.005–.026	Younger

Table S20. Differences in SS between younger (n = 152) and older (n = 83) adults with site 2 excluded and corrected for head motion (fMRI movement). The statistics reported here were calculated using rank-ordered two-way ANOVAs, with age group and sex set as the independent variables and fMRI movement set as a covariate. Ranges of statistics reported here include only values from the indicated significant sparsity thresholds.

Age-group	Cognitive Domain	Network	Sig. Sparsity Thresholds	Correlation (r_s)	Sig. (p)
Younger	Semantic	Global	.05–.50	.175 to .260	.003–.045
Younger	Semantic	DAN	.15–.40	.195 to .220	.012–.026
Younger	Semantic	DMN	.05–.50	.242 to .314	< .001–.005
Older	Executive	Global	.10–.40	.224 to .278	.013–.045
Older	Executive	VAN	.10–.20	.234 to .269	.016–.037
Older	Executive	Limbic	.05–.15	.240 to .285	.010–.032
Older	Executive	DMN	.05–.25	.221 to .322	.004–.049
Older	Semantic	Global	.05–.35	.229 to .317	.004–.041
Older	Semantic	VAN	.05–.20	.238 to .298	.007–.033
Older	Semantic	FPN	.05, .10, .20	.227 to .249	.026–.043
Older	Semantic	DMN	.05–.35	.221 to .310	.005–.049
Older	Episodic	DAN	.05–.45	.237 to .289	.009–.035

Table S21. Cognition remains correlated with global SS and subnetwork segregation following the restriction of the analysis to site 1 (Ithaca; n = 217) and correcting for head motion (fMRI movement). Results are reported as a range of values across all significant (α = 0.05) sparsity thresholds for significant, age, sex and fMRI movement controlled, spearman correlations (r_s) that showed significance for at least three sparsity thresholds. In younger adults (n = 134), semantic cognition was correlated with segregation measures, while executive and episodic cognition were not. Replicating our primary analysis, the effect of segregation was much more widespread in the older group (n = 83), with significant correlations observed for semantic, episodic and executive cognition.

Mediation analysis step	Sparsity Threshold (T)	Effect size (*B*)	Sig.
Age-group → SS (a)	.05–.30	-0.282 to -0.458	p < .001 to p = .042
SS → executive performance (b_1)	.05–.20	0.102 to 0.117	p = .022 to .043
Age-group → SS → executive performance (ab_1)	.05–.20	-0.040 to -0.054	95% CI = [-0.109, -0.012] to [-0.088, -0.003]
SS → semantic performance (b_2)	.05–.50	0.127 to 0.239	p < .001 to p = .040
Age-group → SS → semantic performance (ab_2)	.05–.25	-0.063 to -0.109	95% CI = [-0.135, -0.008] to [-0.201, -0.040]

Table S22. Global SS mediates a negative effect of age-group on cognition. Results are reported as a range of values across all significant (α = 0.05) sparsity thresholds in the PROCESS mediation model. Age Group was a binary predictor set as either young or older and sex, group-relative age, and fMRI movement were set as covariates. Confidence intervals were derived at a confidence level of 95% using 5000 bootstrap samples. When restricting the primary analysis to site 1 (Ithaca; n = 217), the mediation effect of SS on executive performance was lost while the effect on semantic performance became more robust. The total effect of Age-group on semantic performance was positive (B = 0.8690, p < 0.001 in a model with T0.15).
